# The Energy Homeostasis Principle: Neuronal Energy Regulation Drives Local Network Dynamics Generating Behavior

**DOI:** 10.3389/fncom.2019.00049

**Published:** 2019-07-23

**Authors:** Rodrigo C. Vergara, Sebastián Jaramillo-Riveri, Alejandro Luarte, Cristóbal Moënne-Loccoz, Rómulo Fuentes, Andrés Couve, Pedro E. Maldonado

**Affiliations:** ^1^Neurosystems Laboratory, Faculty of Medicine, Biomedical Neuroscience Institute, Universidad de Chile, Santiago, Chile; ^2^School of Biological Sciences, Institute of Cell Biology, University of Edinburgh, Edinburgh, United Kingdom; ^3^Cellular and Molecular Neurobiology Laboratory, Faculty of Medicine, Biomedical Neuroscience Institute, Universidad de Chile, Santiago, Chile; ^4^Motor Control Laboratory, Faculty of Medicine, Biomedical Neuroscience Institute, Universidad de Chile, Santiago, Chile; ^5^Department of Health Sciences, Faculty of Medicine, Pontificia Universidad Católica de Chile, Santiago, Chile

**Keywords:** homeostasis, energy, neuronal networks, behavior, emergent properties

## Abstract

A major goal of neuroscience is understanding how neurons arrange themselves into neural networks that result in behavior. Most theoretical and experimental efforts have focused on a top-down approach which seeks to identify neuronal correlates of behaviors. This has been accomplished by effectively mapping specific behaviors to distinct neural patterns, or by creating computational models that produce a desired behavioral outcome. Nonetheless, these approaches have only implicitly considered the fact that neural tissue, like any other physical system, is subjected to several restrictions and boundaries of operations. Here, we proposed a new, bottom-up conceptual paradigm: The Energy Homeostasis Principle, where the balance between energy income, expenditure, and availability are the key parameters in determining the dynamics of neuronal phenomena found from molecular to behavioral levels. Neurons display high energy consumption relative to other cells, with metabolic consumption of the brain representing 20% of the whole-body oxygen uptake, contrasting with this organ representing only 2% of the body weight. Also, neurons have specialized surrounding tissue providing the necessary energy which, in the case of the brain, is provided by astrocytes. Moreover, and unlike other cell types with high energy demands such as muscle cells, neurons have strict aerobic metabolism. These facts indicate that neurons are highly sensitive to energy limitations, with Gibb's free energy dictating the direction of all cellular metabolic processes. From this activity, the largest energy, by far, is expended by action potentials and post-synaptic potentials; therefore, plasticity can be reinterpreted in terms of their energy context. Consequently, neurons, through their synapses, impose energy demands over post-synaptic neurons in a close loop-manner, modulating the dynamics of local circuits. Subsequently, the energy dynamics end up impacting the homeostatic mechanisms of neuronal networks. Furthermore, local energy management also emerges as a neural population property, where most of the energy expenses are triggered by sensory or other modulatory inputs. Local energy management in neurons may be sufficient to explain the emergence of behavior, enabling the assessment of which properties arise in neural circuits and how. Essentially, the proposal of the Energy Homeostasis Principle is also readily testable for simple neuronal networks.

## Introduction

Throughout evolution, the development of the nervous system has enabled animals with the capacity to manifest ever-growing complex behavior, which has helped them survive in a changing environment. Understanding how neurons arrange themselves into neural networks that work at producing different behaviors has always been a major goal of neuroscience. Various conceptual frameworks have aimed to explain how behavior emerges from neuronal activity. Arguably, the most relevant is the Neuron Doctrine, proposed by Santiago Ramón y Cajal and further developed by Heinrich Waldeyer-Hartz and Horace Barlow (Barlow, 1972; Bock, 2013). Since then, the same logic has spread into coding paradigms (Lettvin et al., 1959; Fairhall, 2014; Yuste, 2015), especially in information processing frameworks (Fodor, 1983; Friston, 2002; Robbins, 2010; Lorenz et al., 2011), and has been scaled from neurons up to neural networks (Yuste, 2015). A common and key element of these conceptual approaches has been to find neuronal correlates of behaviors, effectively associating specific behaviors with distinct neural patterns. This top-down approach (using behavior as a reference to be mapped into neuronal circuits) has been very successful in providing single-unit or network models that can implement the observed behaviors, yet simultaneously, may make difficult the capture of the emergence of behavior, which is by-large a bottom-up phenomenon. This methodological approach also limits our capacity of predicting the boundaries of the capabilities or the spectrum of behaviors of a given system, because we map or associate only those behaviors that have been well-characterized. More importantly, all theoretical approaches, to our knowledge, have only implicitly addressed the fact that neural tissue, like any other physical system, is subjected to several restrictions and boundaries of operations.

Cells use energy to stay alive and at the same time, maintain some reserves to respond and adapt to dynamic situations, maintaining their *homeostasis*. For neurons, energy availability would be further important, as their energy expenses are high, as compared to other somatic cells (Attwell and Laughlin, 2001; Shulman et al., 2004). Indeed, the metabolic consumption of the brain, which represents 20% of whole-body oxygen consumption, contrasts with the neural tissue representing only 2% of whole body weight (Shulman et al., 2004). Interestingly, the total brain energy consumption increases proportionally with the number of neurons among different species, including humans (Herculano-Houzel, 2011), and the total energy expenditure associated to a neuron during the signaling and resting states is constant in different mammalian species (Hyder et al., 2013). Thus, neurons seem to present a highly specialized system for managing their energy demands.

Several evidences demonstrate that it is reasonable to assume a constant value for energy availability for neurons over the long term (energetic homeostasis). For instance, cultured neurons exhibit a steady value for free adenosine triphosphate (ATP) in basal conditions, which transiently decrease during the induction of glutamatergic synaptic activity through various energy challenges (Marcaida et al., 1995, 1997; Rangaraju et al., 2014; Lange et al., 2015). This tight energy management suggests a relevant role for neuronal energy homeostasis on neuronal and network functional properties.

Here, we propose a new bottom-up conceptual paradigm for neuronal networks: The Energy Homeostasis Principle. Under this principle, the condition of maintaining neuronal homeostasis triggers synaptic changes in the individual but connected neurons, resulting in the local energy balance scaling up to a network property. This conceptual framework supposes that energy management might be critical in determining plasticity, network functional connectivity, and ultimately behavior.

## Cellular Homeostasis and Gibbs Free Energy

**Figure 1:**
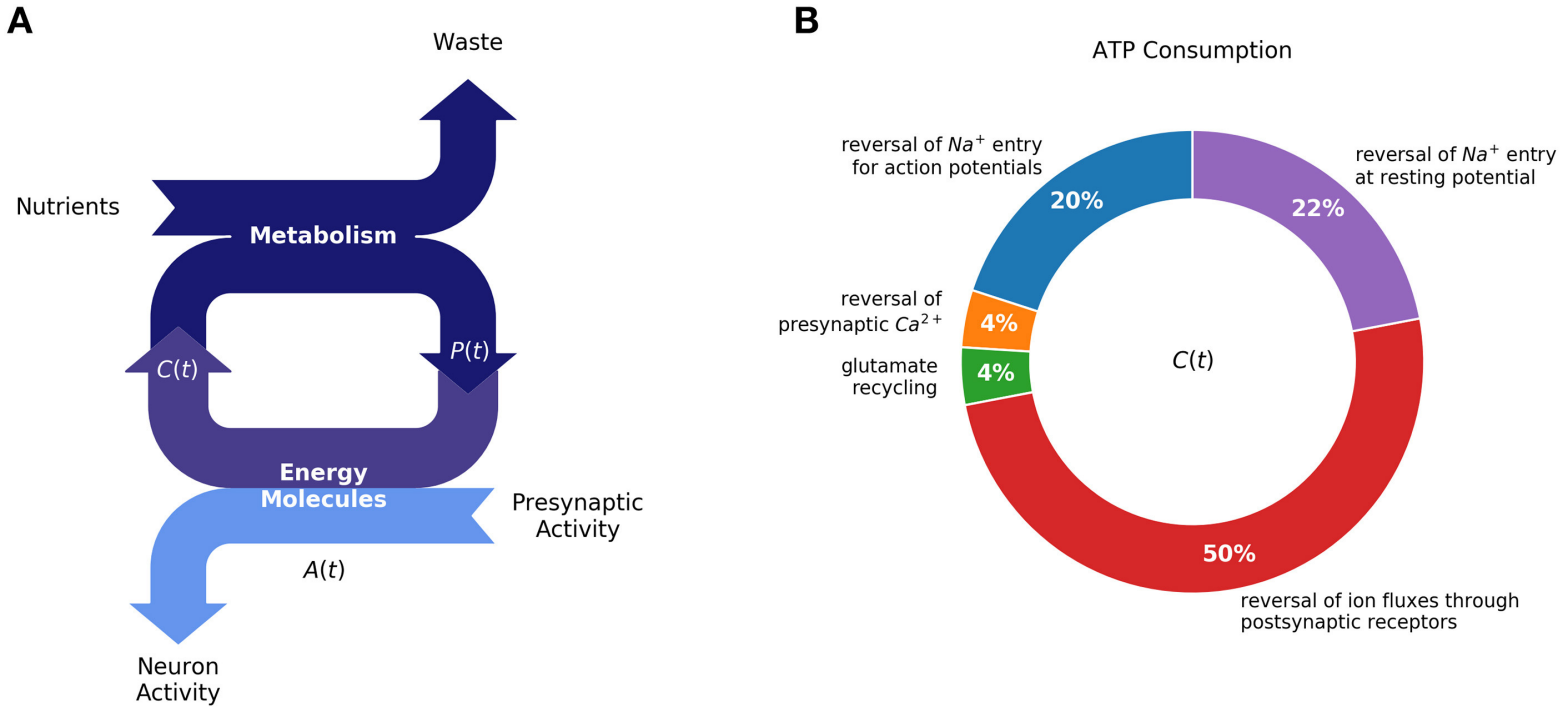
Homeostasis requires a balance between ATP production (metabolism) and ATP consumption (synaptic activity): **(A)** Production of energy molecules by metabolism supports neuron activity, in addition to cell maintenance processes, most notably by ATP [A(t)]. Neuronal homeostasis depends on a balance between production and consumption of high-energy molecules. **(B)** Synaptic activity has been estimated to amount for half of total ATP consumption [diagram redrawn from (Harris et al., 2012)]. For cellular homeostasis to be achieved, neurons must regulate their activity and metabolism in response to changing external perturbations. We propose that regulatory mechanisms, responsible for changes synaptic plasticity, reflect the requirement for maintaining a constant level of energy resources available for neurons to function.

In this article, we propose that behavior may raise as an emergent property rooted in energy requirement of neurons, thus, we would like to start from the level of biochemistry and metabolism. As such, we will begin with the fact that cells are dynamic molecular machines that require the nutrient intake to stay alive. Many biological processes are thermodynamically unfavorable, and through metabolism, cells draw energy from nutrients, and generate metabolic resources necessary to drive their cellular activities (Hofmeyr and Cornish-Bowden, 2000) (for a schematic, see Figure 1A). Cellular homeostasis can be defined as a state where the production and consumption of metabolic resources balance each-other, and thus their concentration is constant in time. For our specific context, balancing the intake and consumption of metabolic resources will unavoidably have a global impact on the cellular processes. The network of metabolic processes is large and complex, limiting, to some extent, our capacity to predict cellular behavior using basic principles. Nonetheless, biochemical reactions must be consistent with the laws of thermodynamics.

Thermodynamics can help us understand how a system evolves in time through the comparison of the thermodynamic potential between an initial and final state. For processes at a constant temperature and pressure, the thermodynamic potential is given by Gibbs Free Energy (G). This thermodynamic potential will dictate a directional bias of chemical reactions. The Gibbs Free Energy—supporting cellular processes—is provided with a finite amount of metabolic resources. Thus, there is a trade-off between the potential for metabolic work and metabolic expenses, which, we propose, may explain some well-established phenomenology of how cells respond to external perturbations. Additionally, the change in the Gibbs Free Energy (ΔG) and the rate associated with chemical transformations are related (Crooks, 1999). To illustrate the relation between thermodynamics and kinetics, for a reversible reaction X⇔Y, the following relation constrains kinetic rates:

(1) $$\begin{array}{r} \frac{rate\left(X\to Y\right)}{rate\left(Y\to X\right)}=e^{-\frac{G\left(Y\right)-G\left(X\right)}{RT}} \end{array}$$

where R is Gas constant, and T the absolute temperature (Cannon and Baker, 2017). This equation describes the relation between the mean rates of any pair of reversible processes (from X to Y, and from Y to X) and the difference in Gibbs Free Energy between the states. Note that by definition the Gibbs Free Energy assumes Temperature to be constant. In simple terms, the difference ΔG{X⇒Y} can be thought of as a “directional bias,” indicating how favorable one direction is over the other. In more detail, the Gibbs Free Energy is divided into two components, Enthalpy (H) and Entropy (S):

(2) $$\begin{array}{l} G\left(X\right)=H\left(X\right)-TS\left(X\right) \end{array}$$

Where, T is the absolute temperature (Silbey et al., 2004). In the context of chemical transformations, Enthalpy is a measure of the energy required to form a given substance, disregarding interactions with other molecules; whereas Entropy can be interpreted as a correction accounting for all possible combinations by which molecules can react (Danos and Oury, 2013). Given the combinatorial nature of entropy, it can also be interpreted as a measure of disorder or information, but certain care must be taken for this interpretation to have physical meaning (Jaynes, 1965). We wish to recognize that the direct application of thermodynamics to biology has many challenges, particularly in describing macro-molecular processes (Cannon, 2014; Cannon and Baker, 2017; Ouldridge, 2018), combining large systems of reactions (e.g., kinetic parameters may be required), and accounting for fluctuations from average behavior (Marsland and England, 2018).

A long-standing observation in biology, rooted in thermodynamic laws is that for cells to function, they must couple unfavorable reactions (ΔG > 0) with more favorable ones (ΔG < 0). Common examples of unfavorable processes are the synthesis of macromolecules, and the maintenance of membrane potential; which are coupled with the hydrolysis of ATP, and GTP providing more favorable ΔG (Nicholls, 2013). In turn, ATP, GTP, and monomers for macro-molecules are synthesized from nutrients through metabolism (Nicholls, 2013). For instance, the maximum free energy provided by ATP hydrolysis is related to the concentration of ATP, ADP, and phosphate.

(3) $$\begin{array}{l} \Delta G\left(ATP\to ADP+Pi\right)=\Delta G^{{}^{\circ}}+RT(\log\left[ADP\right]\\ \;+\log\left[Pi\right]-\log\left[ATP\right]) \end{array}$$

Where, Δ*G*° is the standard free energy, and log, the natural logarithm. For generality, we will call hereafter “energy resources” the set of reactants that allow cells to maintain unfavorable reactions in the direction conducive to cellular functioning and survival. We wish to emphasize that balancing the internal production and consumption of metabolic resources by different reactions is critical, given that metabolic resources are finite and shared by many cellular processes. Thus, cells must manage their internal production and consumption of metabolic resources to stay alive and remain functional, which may be of special consequence to cellular activities with high energy demands, such as synaptic activity in neurons. Given that neurons are active most of the time, it is reasonable to expect that current and future disposal of energy resources is privileged, which may be reflected in the regulatory mechanisms responsible for synaptic plastic changes. In the following section, we will explain how current evidence regarding neuron plasticity appears to support a relatively simple rule: maintain the levels of energy disposal constant, by reducing the consumption of energy resources (e.g., reducing discharge rate, post-synaptic potential), or by increasing high-energy molecule production (e.g., mitochondria and interactions with glia).

## Energy Management of Brain Neurons

Neurons are the paramount example of energy expenditure for their function and survival. This situation is reflected in their large metabolic rates and by the comparatively higher sensibility of brain tissues to oxygen and glucose deprivation (Ames, 2000). Reactions controlling the conversion of nutrients into available cytosolic levels of ATP are important to generate the potential metabolic work that is available to a neuron at any given time. During normal conditions, the primary energy substrate in the brain for neurons is blood-derived glucose; however, when at elevated levels in the blood, ketone bodies and lactate can be used as energy sources as well (Magistretti and Allaman, 2018). The glycolytic pathway is the first step to glucose processing, where two pyruvates and two ATPs are generated from one molecule of glucose. In addition, the pyruvate could either be reduced to lactate or enter the Krebs cycle to produce metabolic reducing intermediates that will generate nearly 29 additional ATP molecules per glucose (through oxidative phosphorylation in the mitochondria). Although neurons and astrocytes are capable of glucose uptake and performing both glycolysis and the Krebs cycle, accumulated evidence supports the hypothesis that neurons may “outsource” glycolytic activity to astrocytes under activity conditions (Weber and Barros, 2015). In addition, the central nervous system is provided with small glycogen reserves, which are predominantly present in astrocytes (Brown and Ransom, 2007), but also found in neurons (Saez et al., 2014). In any case, the lactate derived from glycogen break-down may also provide ATP to the neurons under ischemic or sustained electric activity conditions (Brown and Ransom, 2007).

ATP sources change dynamically with neuronal activity and several mechanisms account for this fine-tuning response. First, neuronal mitochondria are capable of raising ATP synthesis in response to increased synaptic stimuli (Jekabsons and Nicholls, 2004; Connolly et al., 2014; Rangaraju et al., 2014; Toloe et al., 2014; Lange et al., 2015). Although the molecular meditators for this activation are not completely elucidated, the increase of the respiratory rate of an isolated mitochondria correlates well with the ADP concentration (Brown, 1992), and neuronal mitochondrial function has been satisfactorily modeled considering the changes in ATP and ADP levels (Berndt et al., 2015). As an alternative mechanism, it has been reported that operating on milder stimulation conditions, the activity of Na-pump rapidly induces ATP synthesis of the mitochondria, in response to neuronal activity independent from changes in the adenosine nucleotides (Baeza-Lehnert et al., 2018). Second, neuronal activity is known to elicit local increases in blood flow (neurovascular coupling), glucose uptake, and oxygen consumption (Sokoloff, 2008). Coherently, glucose uptake and glycolytic rate of astrocytes are further increased in response to the activity of excitatory neurons, potentially as a consequence of the local rise of glutamate, ammonium (NH_4_), nitric oxide (NO), and importantly, K^+^ (Magistretti and Allaman, 2018). As such, an increased glycolytic rate on astrocytes leads to lactate accumulation that is shuttled into neurons which generate ATP through oxidative phosphorylation. Thus, in CNS neurons, different neuronal and non-neuronal ATP sources work “on demand,” depending on the local levels of synaptic activity.

### What Is ATP Used for in Neurons?

Neurons are perhaps the largest eukaryotic cell in nature, their surface may be up to 10,000 times larger than an average cell (Horton and Ehlers, 2003). The large size of neurons supposes that structural processes, such as protein and lipid synthesis or the traffic of subcellular organelles, should be sustained by high levels of ATP synthesis. In addition to this fact, energy consumption during signaling is far more important. Indeed, it has been estimated that nearly 75% of the gray-matter energy budget is used during signaling; a number that is coherent with the decrease of energy consumption, observed under anesthesia, and is estimated to be around 20% of the total energy budget (Attwell and Laughlin, 2001; Harris et al., 2012).

Most of the neuron's energy budget during signaling is used to restore ion gradients across the plasma membrane, mediated by the action of different ATP-dependent pumps. For example, assuming an average firing rate of 4 Hz, a presynaptic neuron's ATP is mostly used for restoring the Na+ gradient due to action potentials, and to sustain the resting potential (22% and 20% of energy consumption, respectively). Meanwhile, at the post-synaptic neuron, ATP is primarily used to extrude ions participating in post-synaptic currents—about 50% of the energy consumption (Harris et al., 2012). More detailed descriptions of the neuron energy budget is provided in Figure 1B.

### Neuron's ATP Availability Is Tightly Regulated

All cellular organizations require a minimum amount of ATP for survival. It is well-known that when ATP levels decrease below a certain threshold for different eukaryotic cells, apoptosis or necrosis is induced (Eguchi et al., 1997). Nevertheless, determining the maximum and minimum thresholds of a cell's ATP requirement for not only to survive but to realize a specialized function, is less apparent. In any case, this feature must be necessarily shaped by evolutionary adaptations of cells to their specific tissue environment. It is not completely clear how a neuron's ATP levels, during rest and upon activity, may impact its structure and function. Interestingly, by computational and mathematical modeling, it has been proposed that a compromise among energy consumption and information processing capacity has shaped the fundamental features of neuronal structure and physiology, including neuronal body size, ion channel density, and the size and frequency of synaptic inputs (Sengupta et al., 2013). For example, a larger neuronal body has a better capacity to discriminate and respond to different synaptic inputs (coding capacity), but at the cost of higher energy consumption. On the other hand, with a fixed size for the soma, the ion channel density required to obtain maximum energy efficiency is at a lower value than the density needed to maximize the coding capacity. Similarly, although small synaptic inputs at low frequencies are energetically more efficient, better coding capacity arises with larger inputs and rates. These energy constraints may have introduced important consequences during cellular evolution, such that neurons with similar shape and function may harbor similar metabolic features, even across different species.

Remarkably, it has been found that energy consumption of neurons, across the brains of varying species, is constant (Herculano-Houzel, 2011). This result supposes a critical restriction for the function of neuronal networks and their coding properties. For example, sparse coding i.e., brain computations that emerge from the increased firing rate of a few neurons during a task, has been proposed as a mechanistic solution to the limited energy availability for brain neurons (Attwell and Laughlin, 2001; Laughlin, 2001; Lennie, 2003; Weber and Barros, 2015). Thus, it is also possible that variables such as the ATP cytosolic concentration may have been finely tuned during evolution to allow for the emergence of fundamental properties, including some forms of synaptic plasticity.

Accumulating evidence supports that neurons, in time, harbor a narrow window of ATP cytosolic concentration availability [A(t)]. Despite not having dynamic measurements with absolute values of A(t), different experimental approaches on cultured neurons show that this variable tends to remain constant at resting conditions and after momentary synaptic challenges. Accordingly, 60 min of different sorts of glutamatergic stimulation leads to a nearly 5-fold decrease of A(t) (Marcaida et al., 1995, 1997), but when a brief glutamatergic or electric stimulation is applied, only a transient and reversible decrease on ATP levels occurs and the A(t) is subsequently restored to basal levels (Rangaraju et al., 2014; Lange et al., 2015).

Tight management of A(t) also operates on axonal compartments with important functional consequences. For instance, isolated axons from the optical nerve, under low glucose conditions, demonstrate a pronounced decay of ATP levels during high-frequency stimulation (50–100 Hz) (Trevisiol et al., 2017). Interestingly, compound action potentials (CAPs), generated by those stimulated axons, are reduced to the same extent and in high coincidence as the A(t), suggesting that electric activity depends on A(t) (Trevisiol et al., 2017). In addition, isolated axons exhibit a constant value for A(t), which immediately and steeply decays after the inhibition of glycolysis and oxidative phosphorylation, in concomitance with CAPs. However, when inhibitors are washed out, both A(t) and CAPs return to basal levels, further supporting that the system tends to reach a constant value for A(t). The tendency of the system to set a constant value for A(t) is also manifest in conditions where expenditures are highly reduced. For example, A(t) remains constant on pre-synaptic terminals of cultured hippocampal neurons, despite the inhibition of action potential firing due to incubation with the Na^+^ channel blocker Tetrodotoxin (TTX) (Rangaraju et al., 2014). Conversely, the same study showed that electrical stimulation of 10 Hz by 1 min, concomitantly evokes ATP synthesis on pre-synaptic terminals, restoring A(t) to basal levels (Rangaraju et al., 2014). From now on, we will call the basal value of A(t) as the homeostatic availability of ATP (A_H_).

Mechanisms accounting for the intrinsic control of A_H_ in neurons are less explored than in other cells. In the short term, there is a direct and fast effect of ATP molecules and their hydrolysis products, such as AMP/ADP, over the activity of different metabolic enzymes and ion channels. Indeed, neurons are largely known for being extremely, even disproportionately, sensitive to decreases in ATP sources, leading to a fast and significant inhibition of electrical activity (Ames, 2000). For example, ATP-sensitive K^+^ channels open during decreased ATP levels, hyperpolarizing the neuron to reduce endocytosis and the opening of voltage-sensitive Na^+^ channels, thus preventing the ATP expenditure associated to both processes (Ben-Ari et al., 1990). On the other hand, it has been elegantly shown that action potential firing on pre-synaptic terminals' gate activity-driven ATP production is also required to allow proper synaptic transmission (Rangaraju et al., 2014). This close dependency of ATP levels to synaptic functioning has suggested that the affinity constant for ATP (e.g., K_m_) of different pre-synaptic enzymes, might be close to certain resting ATP levels (Rangaraju et al., 2014). It is tempting to speculate that the fine-tuning of the affinity constant from key enzymes might be a broader phenomenon in neurons. In addition, it is known that calcium entry, which is transiently modified by electrical activity, is capable of orchestrating changes in ATP production. For example, synaptic stimulation with brief NMDA pulses, not only lead to pronounced increases of cytosolic calcium levels, but also of the mitochondrial matrix, whose ATP producing enzymes are known to be stimulated by calcium increases (Tarasov et al., 2012; Lange et al., 2015). Indeed, transient increases of calcium levels are thought to be a sort of metabolic alarm which prepares cells to confront high energy demands by increasing ATP production by the mitochondria (Bhosale et al., 2015).

As a complementary mechanism, changes in the ATP and AMP ratio gate the activity of other metabolic sensors which, in turn, induce a specific signaling cascade for short and long-term adaptations of neuronal functions. For example, all known eukaryotic cells, including neurons, harbor energy sensors, such as AMP-activated protein kinase (AMPK), which tend to restore ATP concentration by decreasing anabolic and/or energy consuming processes, while increasing energy production through catabolism post-energy challenges (Potter et al., 2010; Hardie, 2011; Hardie et al., 2012). AMPK is a highly evolutionary-conserved serine/threonine kinase enzyme that is activated either by diminished cellular energy (high AMP/ATP ratio) and/or through increased calcium (Hardie et al., 2012). Recent evidence shows that in dorsal root ganglion neurons—which express the transient receptor potential ankyrin 1 (TRPA1) channel for thermal and pain transduction—the AMPK activation results in a fast, down-regulation of membrane-associated TRPA1 and its channel activity within minutes, which is consistent with lowering energy expenditure by diminishing post-synaptic currents (Wang et al., 2018). Furthermore, it has been demonstrated that calcium overload, induced by an excitotoxic NMDA stimulus on cultured cortical neurons, can be reduced by the activation of AMPK, which would save the energy involved in the reversal of a Ca^++^ potential (Anilkumar et al., 2013). Interestingly, the actions of the catalytic subunit of neuronal AMPK also includes the inhibition of axon outgrowth and dendritic arborization during neuronal development, for adapting to metabolic stress mediated by the suppression of Akt and mTOR signaling pathways (Ramamurthy et al., 2014). This result suggests that AMPK may also operate in mediating structural synaptic changes during the activity of mature neurons, contributing to control energy expenditures in the long-term. Furthermore, it has been shown that the maintenance of long-term potentiation (LTP), which is energetically demanding, is dampened when AMPK activity is pharmacologically activated (mimicking a low ATP/AMP ratio), or conversely, LTP could be rescued when an ATP mimetic, ara-A, was added during an energy challenge. Thus, under low energy conditions, neuronal AMPK tends to inhibit changes on ionic gradients and reduce changes on cytoarchitecture, which can upregulate the value of A(t), impacting plastic capacity as well.

**Figure 2:**
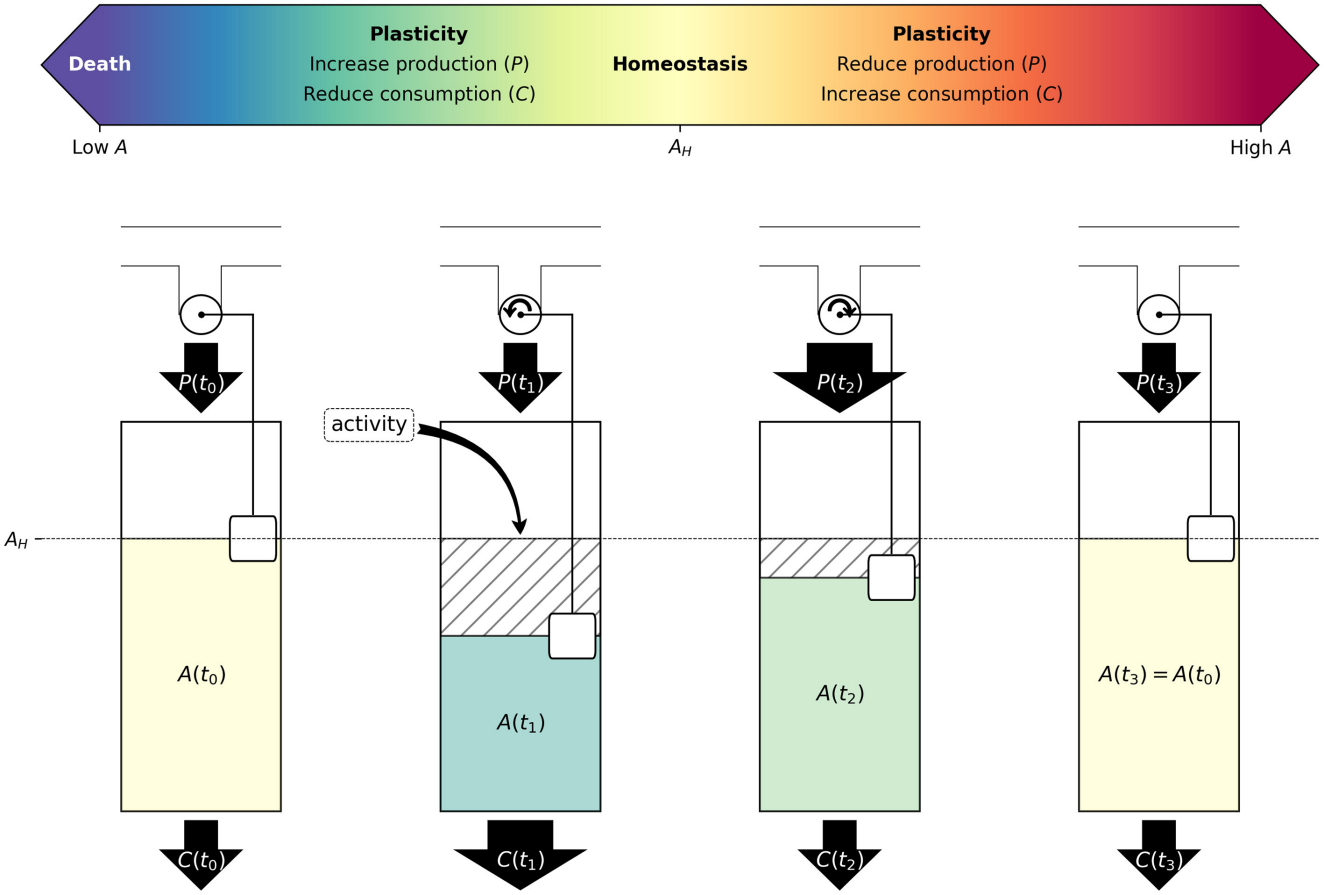
Neuron activity induces changes in metabolism and synaptic activity to maintain homeostatic energy levels: Schematic illustration of neuronal response to an increase in activity. Above, in scale of colors, ATP concentration indicating from low to high A, with A_H_ at the center. Below, step 1 depicts a resting state with A(t_0_) = A_H_. In time step 2, the increase in activity would result in additional ATP consumption, and therefore reduce ATP concentration. We propose that neurons would respond to this perturbation by decreasing ATP consumption [C(t)] and increasing its ATP production [P(t)], as represented in time step 3. Finally, neurons would return to homeostatic levels of ATP (A_H_), which is illustrated in time step 4. During all these steps, A(t) is colored following the above ATP concentration color scale. Some factors that contribute to ATP consumption are as follows: synaptic post-excitatory currents (excitatory inputs), firing rate (excitatory outputs), ion channel density, size of soma, dendrite arborization and axonal length, neurotransmitter recycling and release, and cytoarchitecture adaptations. Whereas, factors contributing to ATP production are: glycolysis supported by glucose or glycogen breakdown, oxidative phosphorylation supported by neuronal pyruvate, astrocyte-derived lactate, and ketone bodies. Neurons possess regulatory mechanisms that sense current energy levels (here represented by a rotating wheel with a floating balloon), and control production and consumption, to maintain homeostasis. Examples of these control mechanisms would include ATP-Sensitive K+ channels, and AMPK signaling.

Summarizing, each neuron has a certain amount of ATP available to them, which is constantly consumed by their different functions which can mostly be explained using ion gradient changes on axons and dendrites. At the same time, ATP production will compensate the ATP expenditure reaching an A_H_ that should remain constant until another specific synaptic challenge arrives (Figure 2). In the next section, we will discuss the potential functional consequences of these adaptations in special cases of neuronal plasticity.

### Revisiting Neuronal Plasticity Under the Perspective of Energy Constraints

A narrow window of ATP cytosolic concentration across time supports a bottom-up view of neuronal energy constraints, which may explain some well-described plastic adaptations from the literature. Measurements of glucose and oxygen consumption (reflecting energy consumption) have not distinguished between the contribution from glial and neuronal metabolism and the total energy expenditure attributed to one neuron (Hyder et al., 2013). Nonetheless, neurons would keep energy availability during the increment of energy demands, which include action potentials, potential propagation or dendritic depolarization, by dynamically sharing expenses with astrocytes glial cells (Hyder et al., 2006; Barros, 2013). It is worth mentioning that energy management is partly performed by these latter cells (Magistretti, 2006; Magistretti and Allaman, 2015). Indeed, we must consider that ATP neural production is provided by the local pyruvate and glial lactate. Where a theoretical model aimed to explain brain energy availability from rat and human brains, it indirectly suggested that glial and neuron lactate sources may dynamically vary across different species and activity levels, with the condition of maintaining a rather constant energy production (Hyder et al., 2013).

We will follow a very simplified view of ATP metabolism characterized by two collections of processes: Those that produce ATP (e.g., from local pyruvate and glial lactate), and those that consume ATP (e.g., recovery of ion gradients, structural and functional synapse maintenance). We can formalize the effect of these processes on ATP concentration (A) by a simply differential equation:

(4) $$\begin{array}{l} \frac{\partial A}{\partial t}=P\left(t,A,\ldots \right)-C\left(t,A,\ldots \right) \end{array}$$

Where, *P*(*t, A*, …) is a function that represents the sum of all reaction rates that produce ATP (e.g., anaerobic and aerobic metabolism), whereas *C*(*t, A*, …) is the sum of all reaction rates that consume ATP (e.g., membrane repolarization, structural and functional synapse maintenance). Both production (*P*) and consumption (*C*) rates are dynamic (they depend on time), but, more importantly, they depend on the levels of ATP available (*A*). Homeostasis will be achieved when production and consumption rates are equal, and the concentration of ATP is constant in time. We will represent the homeostatic concentration of ATP by *A*_*H*_.

We can interpret the observations of relatively constant ATP concentrations in neurons, as reflecting the action of feedbacks that adjust ATP production (*P*) and consumption (*C*) rates, compensating deviations of ATP (*A*), such that neurons return to homeostatic ATP levels (*A*_*H*_). We can expect that in case cells have an excess of ATP, they would respond by decreasing production or/and increasing consumption; and analogously, in case ATP levels are reduced, cells would respond by increasing production or/and decreasing consumption. We will call this regulation the neuron “energy management,” and summarize it mathematically using these equations:

(5) $$\{\begin{array}{c} A>A_{H}\Rightarrow \frac{\partial P}{\partial t}\leq 0,\frac{\partial C}{\partial t}\geq 0\\ A<A_{H}\Rightarrow \frac{\partial P}{\partial t}\geq 0,\frac{\partial C}{\partial t}\leq 0 \end{array}$$

Meaning that the differences between *A* with *A*_*H*_ determines whether ATP production (*P*) and consumption (*C*) processes increase, decrease, or maintain their rates over time. Note that we also consider the possibility that neurons may respond to energy challenges by adjusting production and consumption, but it must be at least one of those variables.

It is critical to notice that this formalization makes some important simplifications. First, we understand that in addition to ATP, the concentrations of ADP, AMP, and other energy resources do determine homeostasis and influence neuronal changes. ATP is a reasonable departure point, given its prevalence in metabolism and the evidence supporting its role in synaptic plasticity, and therefore, will be the main example of energy resource exploit in our argument. An additional reasonable assumption is that the magnitude of the change in reaction rates should correlate with the magnitude of the distance to homeostasis, which we have omitted from the equations but will become relevant later in our argument for proposing experiments. We expect to expand toward a more detailed formalism in future work. Despite its simplicity, we think that our model can help to understand several previous studies and propose some experiments aimed at empirically evaluating the relation between energy resource availability and neural plasticity. We expect that simple phenomenological models, such as ours, will encourage both theoretical and experimental efforts, provided they can be readily falsified empirically, and be compared to theoretical derivations from biochemical first principles.

The tendency to set ATP at A_H_ might be compatible with homeostatic plastic changes that return a neuronal network to a basal firing rate, after prolonged periods of increased or decreased synaptic activity (homeostatic synaptic plasticity). Accordingly, it has been theoretically proposed that the excitability threshold of neurons might be a direct function of ATP (Huang et al., 2007)_._ For example, the K_ATP_ channel-opener diazoxide decreases bursting and regular firing activity of the immature entorhinal cortex neurons (Lemak et al., 2014), which is coherent with a tight association of firing rates with contingent ATP concentration. Also, theoretically, neuronal circuits governed by purely Hebbian-plasticity rules are predicted to converge on instability, or to the opposite—total inactivity (Miller and MacKay, 1994). One possible solution to enable neuronal circuits to remain responsive is to limit the amount of synaptic strength per neuron. At least on excitatory synapses, this problem has shown to be solved by another form of synaptic plasticity termed “homeostatic synaptic plasticity,” and more specifically, “synaptic scaling” (Turrigiano et al., 1998; Turrigiano, 2012). Synaptic scaling emerges to counteract the effects of long periods of increased or decreased synaptic activity in a multiplicative manner, thus allowing neurons to continuously reset the weight of their synaptic inputs to remain responsive to new environmental and cellular contexts. In the long term, the consequence of this regulation is that the firing rate of cortical cells in culture is sustained to an average set point (Turrigiano, 2012). As far as we know, no attempt has been made to relate or prove the influence of neuronal energy load or A(t) on this phenomenon.

Simple experiments on synaptic scaling could be performed to examine whether the tendency to reach A_H_ has a predictive value on the synaptic activity of neuronal networks. As shown in the seminal experiments of Turrigiano's group, when a GABAergic inhibitor bicuculline (Bic) is acutely added to cultured neurons, it produces a significant increase in average firing rate. However, during 48 h of stimulation, firing rates return to control values. On the other hand, neuronal firing rates can be completely abolished soon after adding either tetrodotoxin (TTX) or 6-cyano-7-nitroquinoxaline-2,3-dione (CNQX). Nevertheless, during the 48 h of incubation, activity levels also return to a basal value (Turrigiano et al., 1998). The observed adaptive changes that operate in the long-term makes this experiment ideal for manipulating energy parameters.

**Figure 3:**
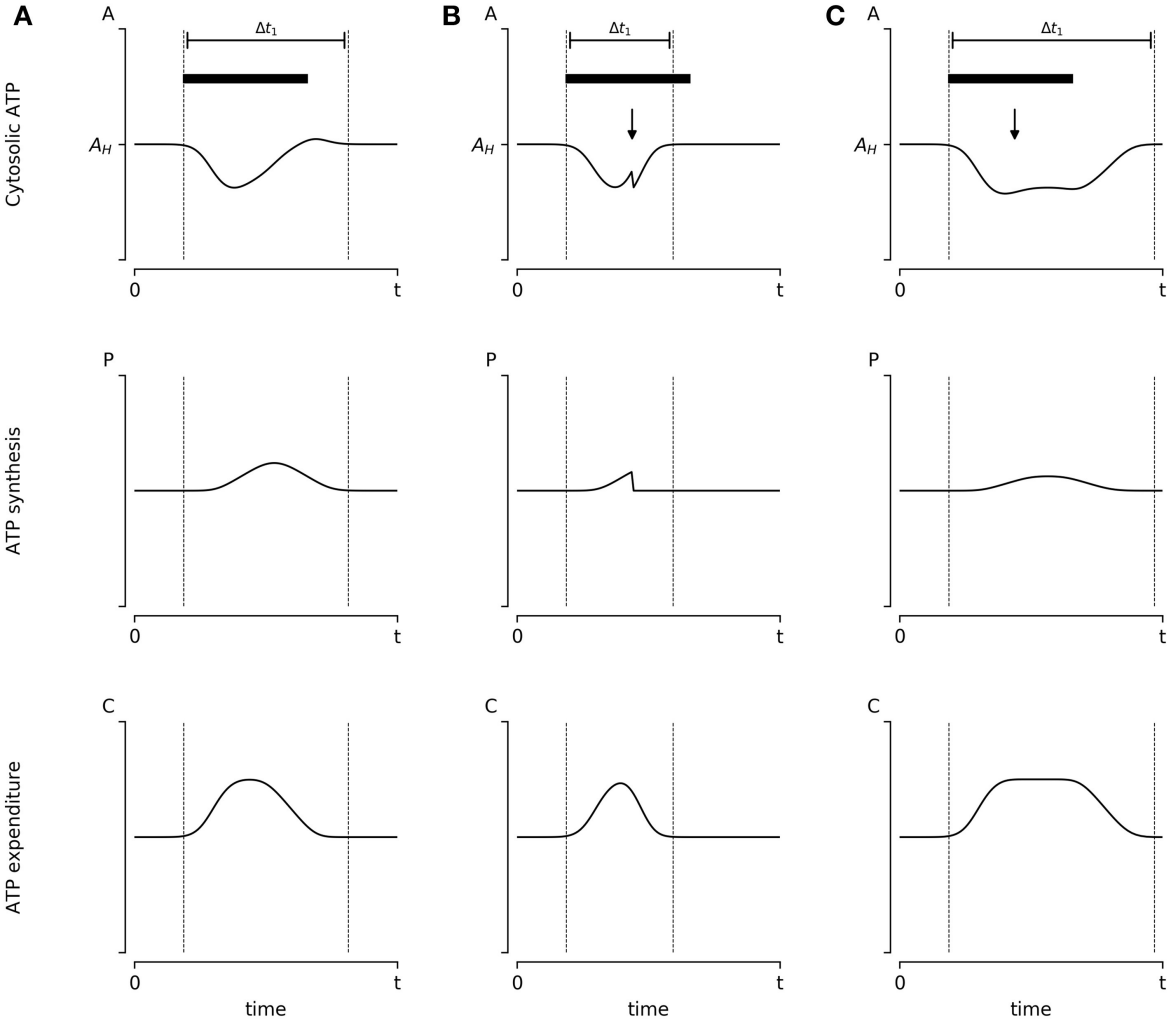
Hypothetical experiment to evaluate the influence of energy availability on synaptic scaling: The influence of energy availability in synaptic plasticity could be evaluated empirically. Here we propose a simple hypothetical scenario and explain what outcomes we predict under each condition. **(A)** Cultured neurons are stimulated with bicuculine (Bic) for 48 h (denoted by a black bar), which presumably induces transient changes in ATP concentration [A(t)]. Neurons respond by increasing ATP production [P(t)] and reducing ATP consumption [C(t)] by reducing its firing rate, which leads to the reestablishment of homeostatic ATP concentration within the time window enclosed by dotted lines. **(B)** During stimulation, cultured neurons can be pharmacologically treated to partially inhibit oxidative phosphorylation (i.e., reducing ATP synthesis) denoted by a black arrow. Following the Energy Homeostasis Principle, we propose this will result in a further reduction of ATP concentration, which will induce an accelerated reduction in ATP consumption through the reduction of synapse firing rates. Thus, we propose that in this scenario the time window required to return to homeostasis is shortened. **(C)** Almost an identical protocol to **(B)** is applied to neurons; however, using an ATP mimetic molecule (denoted by the black arrow). We assume that ATP mimetic molecules would delay the reduction of synapsis firing rate by allosterically inhibiting AMPK, resulting in an enlarged period of energy consumption. Thus, we propose a wider time window before reaching A_H_. All graphics follow Equations 4 and 5, with the additional assumption that the magnitude of the adjustment of P(t) and C(t) are proportional to the distance of ATP levels A(t) to homeostatic levels A_H_. Results from these kinds of experiments could advance the understanding (and potentially manipulate) of the mechanisms responsible for neural adaptations, uncovering the relevant role of metabolic elements, such as metabolic sensors and/or nutrient availability.

Similar to the experiment of synaptic scaling performed by Turrigiano's group, in our theoretical experiment, cultured neurons would be submitted to 48 h of synaptic activity stimulation with Bic. During the stimulus, A(t) will transiently decrease, inducing plastic changes on the network that will return ATP concentration to A_H_, in a given time period (*t*_1_) (Figure 3A). In all conditions, P(t) and C(t) change accordingly with A(t), following Equations 4 and 5. However, if, during the stimulus, the neurons were pharmacologically modified to partially decrease ATP production [e.g., by blocking oxidative phosphorylation with sodium azide], expenditures C(t) are expected to be rapidly lowered and the time window required to return to the A_H_ value will be shortened (Figure 3B). Conversely, one could “enlarge” the theoretical value of A_H_ on cultured neurons by adding an ATP mimetic, such as ara-A. Here we assume that ara-A would cause inhibition of AMPK signaling, and that concentrations employed are low enough not to disturb the ATP synthesis. Thus, we propose that the neurons will take more time to return to A_H_ (Figure 3C)_._ Under these three conditions, the firing rate of neurons should also be adapted to the same level as in the initial state, before stimulation, as well as ATP concentrations should return to the homeostatic value A_H_.

## From Molecules to Behavioral Homeostasis

In the previous sections, we have discussed how the energy homeostasis can affect synaptic plasticity in one neuron. Subsequently, this plasticity can impact other neurons that will trigger the same control systems to keep their A_H_. Since energy demands are transferred through synapses, and synapses appear or disappear according to energy demands, a network homeostasis comes into play. In this section, we argue that energy constraints scale up a level of organization and how homeostasis in one level is affected by homeostasis in the others.

### From Neurons to a Neural Network

The first level is single neuron homeostasis, which is the balance between C(t) and P(t) in single neurons. Importantly, as far as an action potential producing a post-synaptic potential goes, it necessarily imposes an increment in C(t) for the post-synaptic neuron. As such, neurons manage their energy needs which also present an external demand from pre-synaptic neurons, and also imposes an energy demand over the post-synaptic neurons. The fact that a local increase in the C(t) can produce a change in post-synaptic neuron's C(t) supports that energy management is also a neural population property, which we will name network homeostasis. The single neuron homeostasis is closely related to the network homeostasis through a two-way directional interaction, where the network structure imposes constraints on the range of possible homeostatic states that a neuron can achieve, which will, in turn, pose stress on the network through interactions with neighboring neurons. In the same way, these neurons will respond by modifying their synaptic weights (also known as network connectivity), the number and the location of their synapses, thus changing the functionality of the neural network structure (maybe even micro-anatomically). In any condition that causes an imbalance between C(t) and P(t), the neurons will tend to change. Since neurons activate each other through synapses, this means that the activity of the pre-synaptic neurons will induce metabolic work in the post-synaptic ones. In turn, a post-synaptic neuron will modulate its synaptic weight to couple the input from the pre-synaptic neuron to its own metabolic needs. This process will continue recursively until the neurons balance their C(t) and P(t), in which case the network would have reached homeostasis. Essentially, network homeostasis is driven by the needs of each neuron, as each of them will change in an attempt to reach their own A_H_. Note that it is not necessary that every neuron should reach its own A_H_, as the connectivity of activity within the network may not allow them to improve further. However, every single neuron must have enough P(t) to devote toward maintenance processes required to stay alive. As such, network homeostasis becomes a neural population property.

Network homeostasis is tightly related to single neuron homeostasis; therefore, neural network homeostasis will be only achieved when several of the neurons that compose it individually can maintain themselves within homeostatic ranges (e.g., achieving A_H_). It is known that synaptic and dendrite pruning are a part of healthy development (Huttenlocher, 1990; Riccomagno and Kolodkin, 2015), which we could interpret as adjustments required to couple with the trade-off between maintaining the structure vs. the energy spent in action and post-synaptic potentials. In worse cases where suboptimal conditions are imposed on a single neuron by the neural network homeostasis, we expect to find neuron death. This phenomenon is documented as a part of normal brain development in some species (Huttenlocher, 1990), and also in pathological conditions (Perry et al., 1991; Kostrzewa and Segura-Aguilar, 2003; Pino et al., 2014).

### From Neural Networks to Behavior

Behavior can be broadly described as the set of actions performed by an organism, or anything that an organism does that involves movement and response to stimulation. These actions are adaptive when they increase the survival and reproduction probability. In a top-down interpretation of behavior, these actions are the result of the activation of the neuronal circuit that developed evolutionary to fulfill a need. Nonetheless, according to the Energy Homeostasis Principle, at the neural circuitry level, the actions performed by an organism are out of spatial and temporal context, since all the cells experiences are perturbations of the network activity. For a given neuron, the activity dynamics is dependent on the cumulative synaptic currents, regardless of the type of pre-synaptic cells that evoked them, or in the case of sensory receptors, the type of energy that is transduced. Similarly, it makes no difference for a given neuron to have neuron-neuron or neuro-muscular/endocrine synapses. Conversely, we can reinterpret behavior as the observed consequence of the homeostatic activity of an extended neural network (brain) which interacts with the environment. Sensory input and motor outputs can thus be viewed as “environmental synapsis.” Under this framework, what we call behavior may be not necessarily be different from the range of actions neurons engage in any circuit.

However, the interaction with the environment has an important difference that will impact the energy balance in the neuronal network. We can operationalize behavior in a neural system as a set of inputs and outputs that occur in a closed-loop manner. For instance, when we move our eyes, the brain is generating output activity, which in turn modifies the subsequent input to the retina. These dynamics occur for all sensory systems, where motor acts modify sensory inputs (Ahissar and Assa, 2016). In this process, for each brain action, we should expect changes to occur in some sensory inputs. In other words, behavior can be seen as one of the ways in which the brain stimulates itself.

In principle, this closed-loop scheme would enable the brain to completely predict the sensory consequence of the motor actions. This processes of active inference is in line with previous proposals such as Friston's free energy principle and predictive coding (Friston, 2010; Schroeder et al., 2010). It is crucial to note that Friston's Free Energy Principle used an informational approach where aspects such as temperature do not refer to the absolute temperature measured in an experimental setting. As such, the Energy Homeostasis Principle does not conceptually overlap with Friston's proposal; they can be considered as complementary. From a bottom-up view, Friston's proposal answers the epiphenomena, which can be related to information processing, rather omitting the underlying physiological constraints. However, in any of these proposals, there is an agreement that the brain is capable of predicting sensory input, and that it seems to reduce uncertainty as far as possible. In the case of Friston's proposal, it refers to the reduction of informational uncertainty, while in the Energy Homeostasis Principle, it refers to the reduction of energy sensorial input uncertainty.

Parsimoniously, the brain cannot fully predict the sensory inputs that occur after every motor act, because changes that are independent of the action of the organism also occur in the environment, and these changes may be critical to its survival. According to the Energy Homeostasis Principle, we should expect that neural networks will operate in a way that will favor the behavioral input activities within homeostatic energy ranges. If a given input is energetically too demanding, we should expect a change in behavior. If a given set of motor activity consistently produces an energy stressor input, it will cause synaptic changes in the brain, as the energy balance processes are spread over the neuronal network.

**Figure 4:**
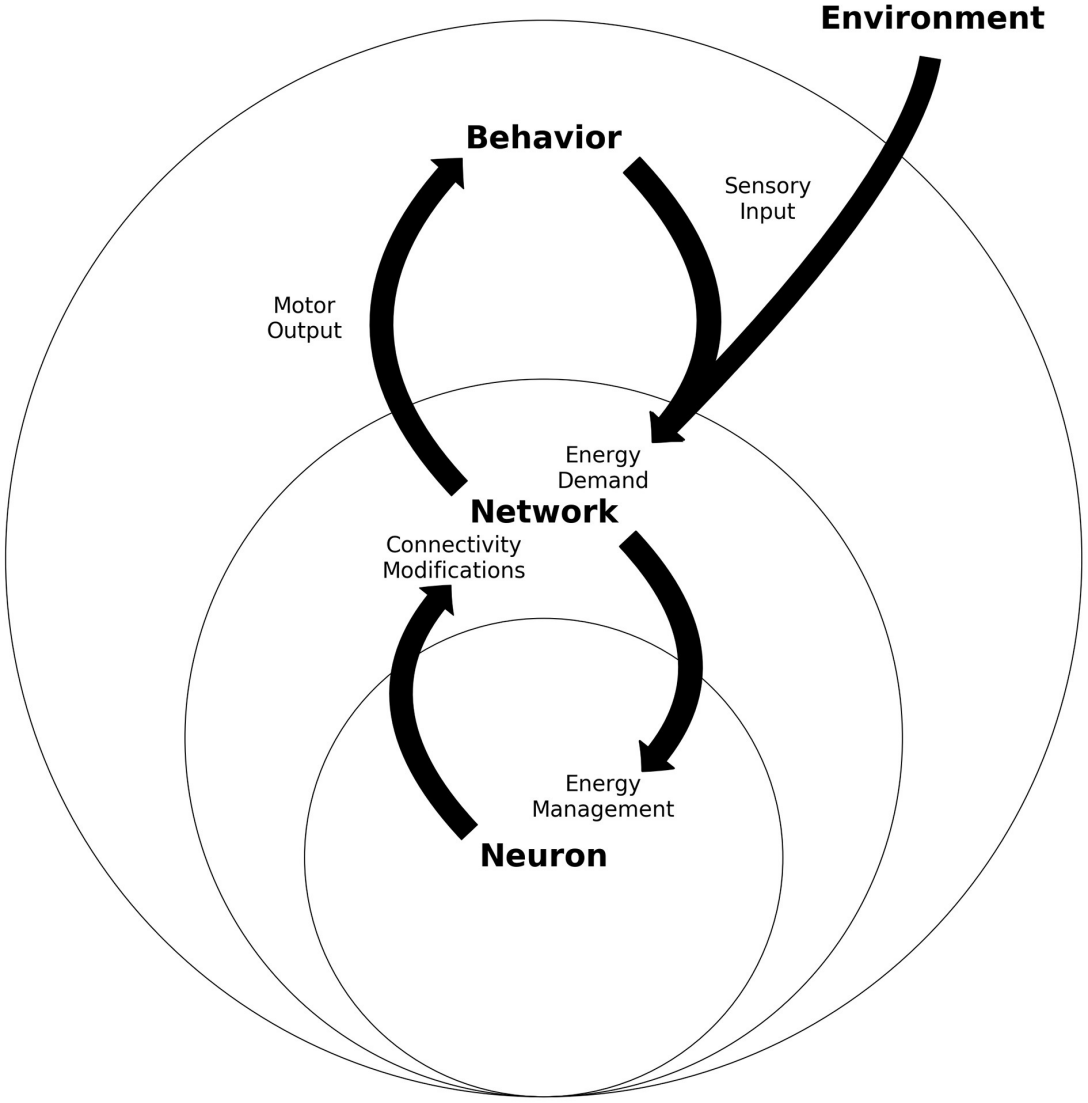
Energy homeostasis: An integrated view of neurons, networks and behavior schematic of the three nested levels of the energy homeostasis system. Each level represents one unit and its proximal operations. Neuron refers to one neuron which must manage its energy consumption, which will trigger neuron-plastic changes. Many of these neurons will build a network, which has connectivity properties and population energy demands. Many networks working together will deploy behavior through motor actions, while also receive the sensory input. All levels present a two-way energy interaction between behavior, neural networks, and neurons. The figure intends to present how sensory input can be considered an energy demand at network and neuron levels, while motor output through behavior gives room to control part of the sensory input.

Sensory input represents the major energy challenge in the brain, while the motor output is the only way a neural network can modify this input. This way, the neuron in the network has the chance to regulate its C(t), given the pressure representing sensory input. Neural networks will restrict the palette of behaviors that can be observed, while behavior will impose energy demands that the neural network will couple with by modifying behavior. For these reasons, behavior can also be considered as a phenomenon which affects the energy homeostasis in a two-way direction. Thus, an at least three-level nested system can be depicted, where each level will have a two-way interaction with each other (see Figure 4).

According to the Energy Homeostasis Principle, a key aspect to explaining adaptive behavior must reside within the brain's macro-structure and evolutionary mechanisms. Given a certain palette of sensory specializations, set of effectors, and brain structures, it will impose the range of possible energy disposal attractors that can emerge. For instance, the number of sensory cells, and their sensitivity to stimuli will determine the energy input imposed on neural tissue. The effectors given will determine the space in which a neural network must control that input. The macrostructure of the brain will then impose general restrictions on the neural network homeostasis. For instance, the visual cortex mainly receives visual input, and the communication through brain regions is mainly achieved using major tracts. As such, the series of C(t), imposed by one neuron on another, must follow the paths allowed by the macrostructure. This means, that neural network homeostasis will not only be a function of energy input provided by the sensory cells, and the chances to control it using effectors, but also of macro anatomical restrictions produced by genetic mechanisms (Gilbert, 2000a,b).

Evolutive pressures must act over all traits—genetic, physiological, and behavioral—of the organism (Darwin, 2003). As such, evolutive pressures have selected a sensory and effectors pallet, as well as a brain macrostructure. We conjecture that from the set of behaviors that satisfy the energy constrictions, behaviors that statistically improve the chances of surviving will be selected. We propose that the macro-anatomical structures impose a certain system of dynamic energy management among neural networks, which force the emergence of a certain set of energy attractors producing, in turn, a specific set of behaviors. It is important to consider that in animals that display a large set of behaviors, probably what is selected is the ability to learn. This concretely would mean that the selected is not a specific behavior, rather the flexibility with which an organism must adapt behaviorally during its own life.

Given this bottom-up view, we conclude the existence of behaviors strictly required for survival, and others which might present adaptive advantages given the specific context of the organism. In human primates, for example, there is a vast set of behaviors that are not strictly for the survival of the single individual in any context, yet they exist, such as leisure activities, those related to the production of art in its multiple forms, and even pathological behaviors which might directly impact the individual's health or survival. As far as these non-strictly adaptive behaviors do not impact the organism's life, they might be highly adaptive in certain contexts.

In any case, evolutionary mechanisms will shape the nervous system's macrostructure and behavior so that both are aligned in a way where solving the energy constraints of a single neuron and the neural network will lead to survival. If not, that macrostructure is expected to be lost, as those organisms will die. In fact, there is no need that all these levels work in alignment per se, rather they must only be aligned to survive. Evolutive pressures will remove the organisms where the three level systems present goals that don't benefit each other. Since we can only see the animals that present all three level goals aligned, we have historically thought that neurons, neural networks, and organisms share the same goal. We proposed here that evolution shaped organisms, so when a neuron solves their needs, the behavior emerges as an epiphenomenon, which enables the organism to solve its needs, hence surviving.

## Perspectives of Reinterpretation

In this section, we aim to contrast our proposal with evidence and highlight the corollary aspects which can open new avenues of research. Concretely, we will evaluate if we can reassess evidence, considering the Energy Homeostasis Principle. We think that this proposal is parsimonious as in spirit the rule is simple, what makes it complex is the wide range of interactions and properties that can emerge from the neural interaction constraint imposed by this rule. We believe that our proposal captures the essence of the concept of Braitenberg's vehicles (Braitenberg, 1986)^1^ and provides a plausible solution to the dynamics elaborated there. Naturally, Energy Homeostasis Principle still has some limitations. It is unclear how we can scale up this principle in networks as large as the brain. The metabolic mechanisms in neurons are quite complex, and still we need more empirical information to tune the mathematical modeling. We decided to use ATP as an energetic proxy, but many other molecules are used by neurons as energetic resources, and may present a dual signaling-resource condition activating control systems. We have mention abundant literature that shows an association between energy related variables and neural activity. However, we have not presented direct evidence of how energy constraints shape plasticity and neural network properties. Despite these limitations, the Energy Homeostasis Principle can be tested empirically by associating plasticity markers with energy availability, production, and consumption as mentioned in section “Revisiting neuronal plasticity under the perspective of energy constraints.” More importantly, this proposal serves new empiric avenues to study the working of the brain. For instance, plasticity has always been thought to be the changes required to fix a given behavior. However, according to our proposal, plasticity is a process that takes place not only during learning but continuously, as a core component of the constant deployment of behavioral changes. As such, plasticity might not only be a determinant of behavior acquisition but a key aspect of ongoing behavior. In the following subsections, we will briefly discuss different strategies which can be used to extend further from Equation (5), an example of evidence interpreted considering the Energy Homeostasis Principle, and then discuss other theoretical and empirical avenues which can be reinterpreted based on this paradigm.

### Modeling Strategies to Implement Energy Homeostasis Principle

We did not extend our mathematical definitions beyond (Equations 4 and 5) as we aim to set a theoretical ground fertile for different modeling strategies. Equations (4) and (5) describe a quite simple idea that neurons take resources to couple with their energetic demands, and that these two must balance each other in order for the neuron to survive. However, the specific strategies used to operationalize the terms within (Equations 4 and 5) was purposefully left open to avoid constrains into specific modeling paradigms. Equations (1–3) were included to better formalize the problem at a metabolic level. These equations are relevant to build the theoretical argument, however, we would not consider them necessary for modeling, at least in a first approach.

In general terms, Energy Homeostasis Principle requires a dynamic modeling, and a topographic or structural component ideally framed from bottom-up. There is already an example that fits with these requirements (Yuan et al., 2018). In this work, they used the ratio between the energy consumed in synaptic transmission and the total metabolic energy consumed in synaptic transmission and dendritic integration over time. This ratio is used as a third component of Hebbian synaptic plasticity, allowing it to change synaptic weighs according to this energetic ratio and pre-synaptic activity. This is a nice example of how to include energetics constraints in neural activity modeling. Under the Energy Homeostasis principle view, the ratio does not make sense in terms of metabolism and neuron needs, because it only address energy consumption, without considering the impacts in productions and availability. Therefore, ignoring the restrictions in energy consumption derived from production and availability. This consumption ratio make sense under a top-down view supported in an information codification logic. Therefore, we suggest to define that ratio according to Equation (4), including consumption, availability, and production following the control mechanisms here presented.

Besides this particular model, graph theory could represent a starting point to define the structure of a dynamic network, in which nodes properties can be updated in a temporal fashion. Graph theory is already used to recall the structural properties of brain networks (Feng et al., 2007; De Vico Fallani et al., 2014), therefore, without a doubt it will be suitable representation which can be extended to consider the energetic management. Moreover, graph theory contains a vast amount of metrics to characterize networks (Costa et al., 2007), and more importantly, could allow to contrast those metrics against real data (Demirtaş and Deco, 2018; Klinger, 2018).

Strategies such as Free Energy Principle (Friston, 2010), or those that profit of predictive coding conceptions (Spratling, 2008; Schroeder et al., 2010; Huang and Rao, 2011), can also serve as a basis for energy homeostatic modeling. However, we suggest to use energy consumption instead of neural activity as predictor. In this case reducing surprise would be analogous to reduce the chances of a neuron to be driven out of energy homeostasis. Energy availability and production ideas are more complex to include. In general terms, and based on the concepts exposed in the previous sections, energy consumption is constrained to energy availability and production. As such, to adapt a predictive coding paradigm requires to include energetic restrictions, which must take into account the rate and amount of energy or activity equivalents that can be managed by the neurons within physiological ranges.

Many other strategies can be used. The above mentioned are often used in neuroscience, however, any modeling strategy that suits the temporal dynamic of energy management, and its topographical bottom-up properties, should be able to capture the essence of the Energy Homeostasis Principle.

### Hybrots: An Analysis Using the Energy Homeostasis Principle

Let us discuss the energy principle proposed here in the context of a simple, *in vivo* network model. Empirically, one critical aspect of relating energy management with behavior is the major challenge of controlling sensory inputs. Most of experimental, *in vivo* animal models are not only sensible to acoustic, visual, physical, and chemical stimuli of the environment, but also to proprioceptive inputs, such as muscle contraction, tendon tension, blood acidification, hormone levels, among others. Strictly speaking, there is no way to properly control the sensory input of an animal *in vivo*, and the behavioral *in vitro* protocol seems to be unreal. Nonetheless, there are some protocols that can be considered as initial efforts of trying to build *in vitro* behavioral protocols. Specifically, some reports demonstrate that if we connect a neuronal culture of dissociated cortical or hippocampal neurons to an external device, coherent behavior can be obtained (Novellino et al., 2007; Tessadori et al., 2013).

Concretely, a system decodes the firing rate of the neurons in the culture and generates an output which is used to control two wheels of a vehicle. The vehicle has distance sensors. The sensor activity is coded to electrical pulses delivered back to the culture. The stimulation frequency is a function of the distance to an obstacle in front of the sensors. If the vehicle gets closer to an obstacle, then the stimulation frequency increases. If the vehicle crashes into an obstacle, a stimulation (20 Hz for 2 s) is delivered, which is previously known to trigger plasticity (Jimbo et al., 1999; Tateno and Jimbo, 1999; Madhavan et al., 2007; Chiappalone et al., 2008; le Feber et al., 2010). Leaving the vehicle in a circular maze with several obstacles under the operation of this protocol will cause it to “learn” to navigate, while avoiding impacts with obstacles (Tessadori et al., 2012). This model constitutes a protocol that enables studying the molecular, electrophysiological, and behavioral properties of neural processing simultaneously; above all, it allows the full control of the sensory input that this network will have.

**Figure 5:**
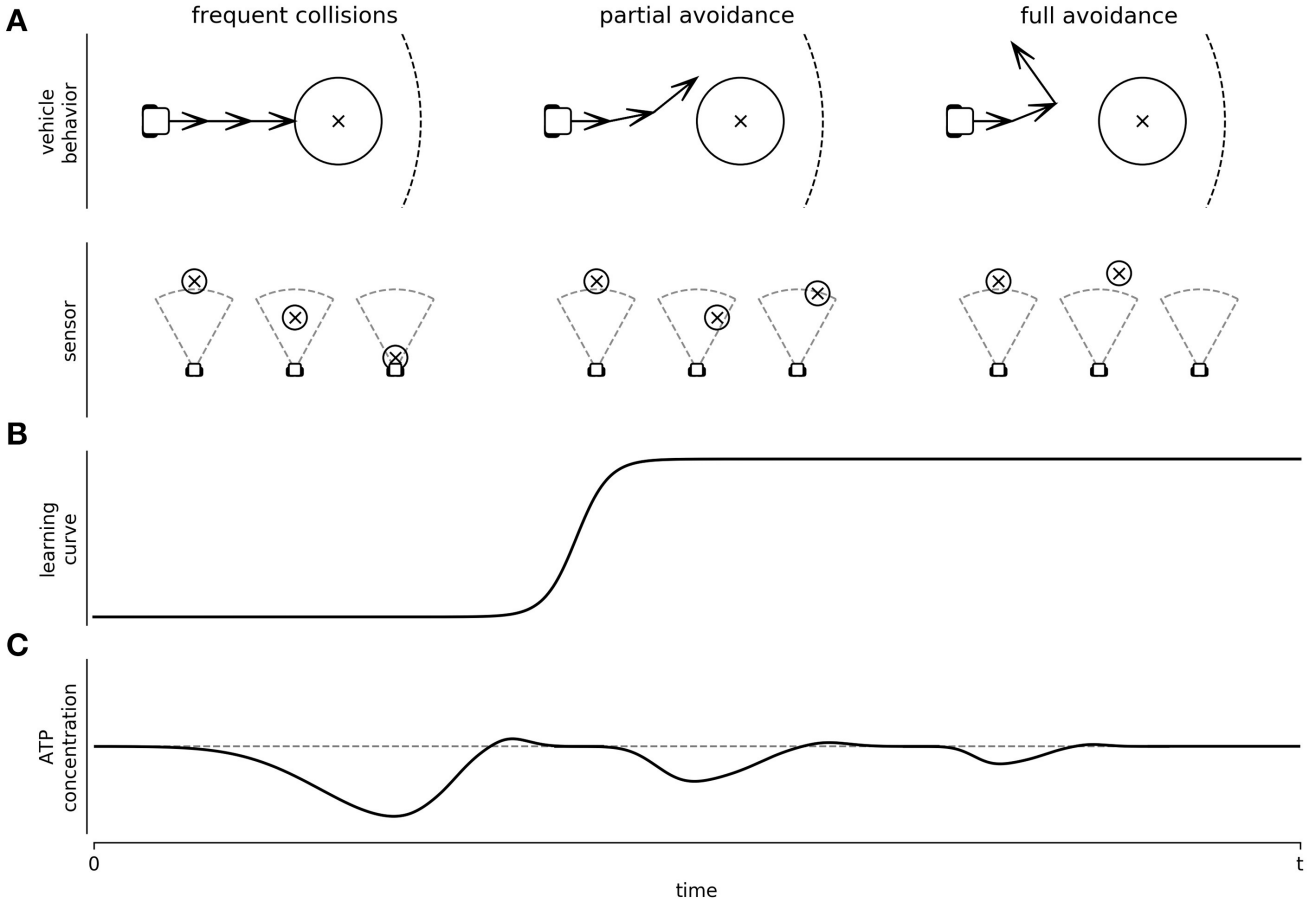
Depiction of Braitenberg's vehicle behavior as a controlled platform to study learning and network energy adaptation: In this figure we hypothesize, based on the Energy Homeostasis Principle, how a hybrot would learn. **(A)** Different behaviors observed at different learning levels of the Braitenberg's vehicle (top panel), and the corresponding sensory input (bottom panel) from an obstacle (circle centered to x symbol) is detected by a sensor (dashed area), while the vehicle explores the environment. **(B)** Learning curve of the vehicle when learning to avoid the obstacles, passing from frequent collisions to full avoidance behavior. **(C)** Network energy adaptation triggered by the sensory input while minimizing the energy stress.

Is this learning-like phenomenon compatible with the Energy Homeostasis Principle? When a single neuron is submitted to constant stimulation, we expect to have a 1–1 stimulation-action potential response. However, at a frequency stimulation as low as 10 Hz, the neurons will decay over time until they are unresponsive, or their response is importantly delayed (Gal et al., 2010). If interpreted through the Energy Homeostasis Principle we can hypothesize the following mechanism. First, we can postulate that at a frequency of 10 Hz or higher, stimulations become energetically stressful. As a response, neurons will respond with modifications in their synaptic weights in the short term, and with changes on their cytoarchitecture in the long term. Both processes will result in changes to the network structure. Each time the vehicle crashes, a stressful 20 Hz pulse will be delivered inducing plasticity. Functional restructuration is expected at each impact; leading to a random walk through different neural functional configurations, where each neuron will jump from state to state to minimize energy stress (see Figure 5). It is expected that those network configurations that decrease the effects of the sensory input will reduce energy stress due to impacting obstacles. But the best network configuration to the energy stress is indeed to avoid it. Eventually, a network configuration will arise which will prevent the vehicle from crashing. Since no energy stress will be delivered as a sensory input with this configuration, this structure will seemingly stabilize on a configuration of homeostatic energy expenditure (Figure 5). We are aware that the above interpretation may oversimplify the actual mechanisms followed by the neurons. Neuronal changes are most likely not completely random and more complex regulations may be taking place. However, we want to point out that they can be sufficient to explain the phenomenology of the observations. As such, energy management, as a local rule, will impact the neural network structure as an emergent property, where, in turn, it will impact behavior. Critically, in this example, we have focused on sensorial input as an increment of neural activity. This might not always be the case (such as under sensorial isolation). Despite that, under this specific scenario, we propose that networks will minimize energy consumption; the goal is to arrive to A_H_, not to the minimum possible energy expenditure. Therefore, if the sensorial input would move A(t) below A_H_, we would expect network modifications to increase expenses. In any case, the obtained behaviors must be at least compatible with the dynamic constraints imposed by C(t), despite it being too high or low. In this example, behavior emerged to satisfy the energy needs of the neuron by means of C(t). Finally, from all the vehicle movements, only a few, like avoiding the obstacle, might be interpreted as purposeful from an observer's point of view, the remaining ones may be considered a random trajectory. Importantly, this attribute is provided by the observer, as the neurons would only be managing their energetic demands. More research is required to evaluate what is happening with *behavior*, when the obstacles are out of the sensor's range along with the *learning* curve of the vehicle. Nonetheless, the Energy Homeostasis Principle allowed us to propose this hypothesis (Figure 5C), and it can be empirically addressed. Naturally, using the same experimental approach, we can evaluate how plasticity is affected by energetic demands induced electrically or by altering neurotransmitter concentrations. We can use the vehicle's behavior, or we can use the Graph Theory index already used to characterize networks (Costa et al., 2007) to associate neural network properties with energetic demands and metabolic activity.

### The Neuron Doctrine and the Energy Homeostasis Principle

Historically, the primary efforts to connect neuron activity with neural network dynamics and behavior was first proposed in 1888 (Barlow, 1972; Bock, 2013), which is referred to as “The Neuron Doctrine,” maintained and developed to this day (Dehaene, 2003; Moser and Moser, 2015; Sheppard, 2016). In general terms, this theoretical proposal tries, in dual form, to solve the information coding and processing problem and has been supported by intracranial recordings, where abundant examples can be found (Lettvin et al., 1959; Fairhall, 2014; Moser and Moser, 2015). Specifically, neurons are expected to code for specific properties of the environment, where its activity is associated with the detection of specific stimuli. For instance, neurons in the primary visual cortex of mammals are selectively sensible to oriented bars (Hubel and Wiesel, 1962; Taylor, 1978), while in the Lateral Geniculate there is evidence supporting the existence of circular receptive fields representing portions of visual space (Reid and Shapley, 1992). In these cases, neurons have receptive fields, which can be interpreted as a specific topologic relation of V1 with a certain retinal region; and therefore, with the image. Receptive fields with the same selectivity feature can also be found in the tactile, and auditory primary cortex, evidence of which is often interpreted as environmental stimuli being coded as a map in the brain (Penfield and Boldrey, 1937; Penfield, 1965; Ruth Clemo and Stein, 1982). This classic evidence is also theoretically line up with the recent hippocampus where neurons (Moser and Moser, 2015).

Critically, most of the evidence supporting the neuron doctrine is associated to the neuron discharge rate. Since this discharge rate is a part of the C(t), it necessarily means that most of the evidence supporting the neuron doctrine supports the Energy Homeostasis Principle as well. For this reason, it is plausible to consider that most of the neuron doctrine evidence is also evidence indicating how energy expenses of one neuron can be directly associated with behavior. Furthermore, high discharge rates, as mentioned above, are expected to trigger plasticity mechanisms. Also, only a low percentage of neurons present high discharge rates (Olshausen and Field, 2005), which should be expected under the Energy Homeostasis Principle scope. Moreover, due to the fact that high discharge rates might trigger changes in functional connectivity (synaptic weights), it should not be surprising that when presenting more complex visual scenes, classic receptive fields are no longer detectable (Fairhall, 2014). We may consider that classic stimulation visual protocols impose an energy input, reflected in the high discharge rate, which needs to be managed. In contrast, visual scenes are regularly experienced, therefore already managed energy, and the firing pattern are considerably lower. As such, we think that the neuron doctrine is not necessarily wrong, rather it has not focused on how the discharge rate is a proxy of energy demands imposed on neurons, which in turn affects their homeostasis. Also, that plasticity might have a functional role in ongoing behavior rather than only stabilizing learned behaviors.

The neural doctrine paradigm has been closely related to information coding paradigms. The coding paradigms follow the same logic as the genetic code; the idea that information is universally coded using the same dictionary or codebook. In the case of genetics, what we call the genetic code, is an arrangement of how the sequence of nucleic acids informs specific amino acid sequences when assembling proteins. In the case of a neural code, the assumption is that environmental stimuli are translated into brain activity, which is then translated into motor output. More specifically, it is possible to map specific neuron activities to specific properties of the environment. For instance, the intensity of the stimulation can be mapped to the discharge rate of the sensory neurons (Gardner and Johnson, 2013). The transduction of the stimuli is usually non-linear and sensitive to differences with previous stimulation rather than the raw value of the stimuli—Weber's law (Gardner and Johnson, 2013). This adaptation law has a direct interpretation in the context of energy expenditure by neurons, as neurons coding raw stimuli would demand a greater energy supply. Weber's law has also been extended to complex cognitive functions, such as quantity estimations (Dehaene, 2003)—where discharge rates are used as the code of quantities for specific neurons—suggesting that energy saving may be a strategy widely used by neurons.

Of course, the discharge rate is far from being the only neural code proposed. Coupled with the complexity of sensory activity, temporal coding was proposed, where the exact temporal relationship between each neuron spike would be the key to understanding how environmental information is translated into brain activity (Connor and Johnson, 1992; Friston, 1997). Temporal coding is implicitly related to energy demands, as the time between action potentials trigger plasticity mechanisms, associated with one of the most expensive items of the neuron physiology—post-synaptic potential and plastic mechanisms (Attwell and Laughlin, 2001). Another strategy was population coding (Georgopoulos et al., 1986; Nicolelis, 2003; Moxon and Foffani, 2015). Population coding uses the activity of a high number of neurons, where the discharge rate, timing, and as many properties can be extracted make it possible for a human or non-human primate to move a robotic arm or similar, with the brain. As more neurons are included, more information is obtained, and we should expect that we will better predict the arm movement. This approximation is good when the aim is to predict behavior but is not useful to understand how behavior emerges from neural activity. If reassessed using Energy Homeostasis Principle, we interpret that population coding works as it is a good assessment of neural network homeostasis, implicitly providing information about plastic changes and neural energy management. Up to some extent, all approaches have to do with when, how much, and which neurons are discharging, which in turn can be interpreted as when and how much energy is expended by individual neurons and the network.

When evaluating evidence related to a whole-brain approach, the neuron doctrine is mostly applied by associating the bold signals of brain regions to specific behaviors. Critically, the fMRI signal is derived, to some extent, by the changes triggered through the glia to couple with the energy demands (Otsu et al., 2015). Therefore, we can interpret that energy management associated to glial function, is already associated directly with behavior. Moreover, it suggests that energy management can be mapped into networks associated to specific behaviors. Naturally, the specifics in which Energy Homeostasis Principle would impact large networks like brains is still elusive, and it probably would require to incorporate formally the functional properties of the glia.

In general, the fMRI approach strongly resembles the serial symbolic programming paradigms, where a module can be homologized to a programming function, and the network would be the general architecture of the software. The loss of a programming function leads to the loss of a specific functionality of the software. This metaphor was addressed in classic literature (Hovland, 1960; Searle, 1980), suggesting that the brain processes information using a symbolic serial paradigm. As such, most of the neural correlates within the neurocognitive domain are interpreted as information processing, ranging from a strictly symbolic to a correlative information approach. However, using a bottom-up approach and the Energy Homeostasis Principle, those attributions are an observer's bias, as the one described in Braitenberg's vehicles (Braitenberg, 1986). Behavioral functions of a neuron or the neural network would be the epiphenomena of neurons regulating their own homeostasis. In fact, as explained in the previous section, we can describe how the vehicle learns to avoid obstacles without using any informational, symbolic, or teleological explanations. Using this bottom-up approach, it is expected that an informational approach will be useful, as far as the neurons' and the neural network's needs are aligned with the organism's. However, it should be interpreted as an epiphenomenon of neural networks solving their own needs.

### Reinterpreting Evidence Toward New Research Avenues

As we have discussed above, energy management, though implicitly considered, is a key feature of the nervous system. This necessarily means that most of our current evidence can be reinterpreted in the light of the Energy Homeostasis Principle. We expect that this reinterpretation will trigger new ideas and strategies to understand the neural phenomena. As an example, we may try to explain the neuronal changes associated with learning processes, based on iconic paradigms such as the long-term potentiation (LTP) and depression (LTD) (Nabavi et al., 2014; Jia and Collingridge, 2017). Both phenomena involve a large amount of energy expense where the ATP could be followed to understand the phenomena of plasticity as one of energy management. This is key, considering that even the Hebbian rules (Kempter et al., 1999), operates differently, according to the neuron type (Abbott and Nelson, 2000), highlighting the difficulties in predicting plasticity according to neural activity. At the same time, the calcium ion plays a critical signaling role within neural physiology, where we should ask if it might be a signal of energy expenditure. It is known that metabolic processes sense the ATP-AMP ratio (Ames, 2000), however, they have not been studied in association to the plasticity phenomena.

Consequentially, we can assess energy management and not solely from a molecular or electrophysiological perspective. For instance, can we consider inhibitory neurons as an adaptive feature to control brain energy expenditure? This is most intriguing if we consider that inhibitory neurons are key to increasing the neural circuits' controlling properties (e.g. negative feedback structures).

Simultaneously, the central nervous system is the only structure of the body which is actively isolated from the vascular system. It has its own system to maintain stable the neuron proximal environment. Moreover, astrocytes coordinate themselves through calcium waves, producing local changes in blood flow and hyperemia (increase on blood irrigation) (Otsu et al., 2015). The brain-blood barrier is not only a filter, but it works functionally to support the energy demands of the neural networks. In fact, synapses are currently suggested as tripartite structures (neuron-neuron and astrocyte) (Wang and Bordey, 2008), where the glutamate-release excitatory synapses are proposed to control neurovascular coupling, and thus, brain energy during conditioning and behavior (Robinson and Jackson, 2016). This would be a clear example of a neural activity involving external support for energy management.

Moreover, there is a vast number of shapes for neural cells. It is currently unknown why some neurons display large, dendritic arborizations and short axons, while others present long axons and rather small dendritic arborizations. Similarly, there are varying basal discharge rates of activity. We think it is worth exploring whether the likelihood of particular morphologies and rate of activities are associated with energy constraints. For instance, can a neuron manage to maintain a long axon and at the same time a huge dendritic arborization where it must maintain a large number of dendritic spines? If we explore the evidence we already have, we are confident that new insights into neuron morphology will appear. Even more, if an unlikely neuron shape or size which is energetically more expensive presents itself; we should expect that those neurons would be more sensible to energy demands and may be more susceptible to neural death (Le Masson et al., 2014). In fact, Paul Bolam proposed that the reason behind Parkinson's is due to the dying out of dopaminergic neurons because of their huge size, which is very expensive in energy terms (Bolam and Pissadaki, 2012; Pissadaki and Bolam, 2013). It is most likely that many of these traits are genetically determined, however, energy constraints might limit the possible morphological variety. Furthermore, that genetic determinants of neuron specializations may be triggered in response to the C(t).

Finally, the Energy Homeostasis Principle paradigm, combined with a bottom-up view, allows us to reinterpret behavior in a much more flexible way. Animals display many behaviors that are not intrinsically adaptive. Leisure activities are an evident example. Why the dog likes to go for the ball or follow a car? Why would we like to learn how to play the piano or to paint? Using a top-down approach would force us to interpret that evolution endorses us with a leisure activity brain module and that all behaviors are somehow beneficial. It seems more parsimonious to think that evolution restricted the system through macrostructure, so that survival-related brain functions will be selected and inherited. Above all, a wide set of diverse, seemingly useless behaviors can appear, without compromising organism survival or neural needs. Therefore, the only constraint for behavior is that the organisms must stay alive and that the sensory input can be successfully managed, in terms of its energy demand, by the neural networks and the neurons within them. As we already explained before, we think that in the cases of the vehicles controlled by neural cultures, the rules of the stimulation given is critical in understanding how they learn to avoid obstacles. From all the works that reported learning-like properties of *in vitro* dissociated cultures of neurons (Novellino et al., 2007; Mulas and Massobrio, 2010; Tessadori et al., 2012), two main conclusions can be obtained: (1) learning-like properties are not dependent on a priori, highly intricate and sophisticated neural structures, and (2) there is at least one property which does not require a brain evolution argument to explain the emergence of behavior (but probably requires a neural tissue evolution argument). This would be particularly important in relation to behaviors that are not directly tied to survival.

Because of the space limitations, many of these latter considerations are laid out in a basic form. Nonetheless we stress that some of these speculations can be assessed by reviewing the current literature under the Energy Homeostasis Principle rationale. However, the proposal may encourage the development of falsifiable hypotheses, allowing for the testing of these intuitions through empiric work. Therefore, we propose the principle as a novel paradigm from which we can reinterpret neuroscience experimental data, as well inspire the design of experiments which may connect biochemical knowledge to cognitive neuroscience.

## Author Contributions

RV, SJ-R, and AL developed the initial general argument. SJ-R was the main contributor to the metabolic and biochemistry sections. AL was the main contributor to the single neuron's physiological sections. RV was the main contributor to the neural networks and behavior sections. PM contributed by articulating the different sections to build the manuscript. RV wrote the first draft. CM-L designed and built all the figures, based on all the authors' recommendations. CM-L, AC, and RF contributed with key observations that refined the general argument. All authors revised and edited the manuscript. RV and PM edited the final version.

### Conflict of Interest Statement

The authors declare that the research was conducted in the absence of any commercial or financial relationships that could be construed as a potential conflict of interest.

## Footnotes

^1^Braitenberg vehicle is a concept proposed by Braitenberg (1986), referring to a simple vehicle that has two sensors, each connected to an effector, i.e., a wheel, that provides the vehicle the ability to navigate in a given environment. The activation of the sensors can increase or decrease the speed of the respective wheel. In addition to the sensor-effector relationship, the physical configuration of the sensors and the wheels will determine the navigation “behavior” of the vehicle when the stimuli are present in the environment. One major conclusion of this concept is that complex behaviors arise from relatively simple properties (sensors, circuitry, and effectors) of the system in interaction with the environment.

## References

[B1] Abbott L. F. Nelson S. B. (2000). Synaptic plasticity: taming the beast. Nat. Neurosci. 3, 1178–1183. 10.1038/81453 11127835

[B2] Ahissar E. Assa E. (2016). Perception as a closed-loop convergence process. Elife 5, 1–26. 10.7554/eLife.12830 27159238 PMC4913359

[B3] Ames A. (2000). CNS energy metabolism as related to function. Brain Res. Brain Res. Rev. 34, 42–68. 10.1016/S0165-0173(00)00038-2 11086186

[B4] Anilkumar U. Weisová P. Düssmann H. Concannon C. G. König H. G. Prehn J. H. M. (2013). AMP-activated protein kinase (AMPK)-induced preconditioning in primary cortical neurons involves activation of MCL-1. J. Neurochem. 124, 721–734. 10.1111/jnc.12108 23199202

[B5] Attwell D. Laughlin S. B. (2001). An energy budget for signalling in the grey matter of the brain. J. Cereb. Blood Flow Metab. 21, 1133–1145. 10.1097/00004647-200110000-00001 11598490

[B6] Baeza-Lehnert F. Saab A. S. Gutiérrez R. Larenas V. Díaz E. Horn M. (2018). Non-Canonical control of neuronal energy status by the Na^+^ pump. Cell Metab. 29, 668–680.e4. 10.1016/j.cmet.2018.11.005 30527744

[B7] Barlow H. B. (1972). Single units and sensation: a neuron doctrine for perceptual psychology? Perception 1, 371–394. 10.1068/p010371 4377168

[B8] Barros L. F. (2013). Metabolic signaling by lactate in the brain. Trends Neurosci. 36, 396–404. 10.1016/j.tins.2013.04.002 23639382

[B9] Ben-Ari Y. Krnjević K. Crépel V. (1990). Activators of ATP-sensitive K+ channels reduce anoxic depolarization in CA3 hippocampal neurons. Neuroscience 37, 55–60. 10.1016/0306-4522(90)90191-6 1978742

[B10] Berndt N. Kann O. Holzhütter H. G. (2015). Physiology-based kinetic modeling of neuronal energy metabolism unravels the molecular basis of NAD(P)H fluorescence transients. J. Cereb. Blood Flow Metab. 35, 1494–1506. 10.1038/jcbfm.2015.70 25899300 PMC4640339

[B11] Bhosale G. Sharpe J. A. Sundier S. Y. Duchen M. R. (2015). Calcium signaling as a mediator of cell energy demand and a trigger to cell death. Ann. N. Y. Acad. Sci. 1350, 107–116. 10.1111/nyas.12885 26375864 PMC4949562

[B12] Bock O. (2013). Cajal, Golgi, Nansen, Schäfer and the neuron doctrine. Endeavour 37, 228–234. 10.1016/j.endeavour.2013.06.006 23870749

[B13] Bolam J. P. Pissadaki E. K. (2012). Living on the edge with too many mouths to feed: why dopamine neurons die. Mov. Disord. 27, 1478–1483. 10.1002/mds.25135 23008164 PMC3504389

[B14] Braitenberg V. (1986). Vehicles: Experiments in Synthetic Psychology, 2nd Edn. Cambridge: MIT Press, 168.

[B15] Brown A. M. Ransom B. R. (2007). Astrocyte glycogen and brain energy metabolism. Glia 55, 1263–1271. 10.1002/glia.20557 17659525

[B16] Brown G. C. (1992). Control of respiration and ATP synthesis in mammalian mitochondria and cells. Biochem. J. 284, 1–13. 10.1042/bj2840001 1599389 PMC1132689

[B17] Cannon W. R. (2014). Concepts, challenges, and successes in modeling thermodynamics of metabolism. Front. Bioeng. Biotechnol. 2:53. 10.3389/fbioe.2014.00053 25505786 PMC4244978

[B18] Cannon W. R. Baker S. E. (2017). Non-steady state mass action dynamics without rate constants: dynamics of coupled reactions using chemical potentials. Phys. Biol. 14:055003. 10.1088/1478-3975/aa7d80 28675379

[B19] Chiappalone M. Massobrio P. Martinoia S. (2008). Network plasticity in cortical assemblies. Eur. J. Neurosci. 28, 221–237. 10.1111/j.1460-9568.2008.06259.x 18662344

[B20] Connolly N. M. C. Dussmann H. Anilkumar U. Huber H. J. Prehn J. H. M. (2014). Single-cell imaging of bioenergetic responses to neuronal excitotoxicity and oxygen and glucose deprivation. J. Neurosci. 34, 10192–10205. 10.1523/JNEUROSCI.3127-13.2014 25080581 PMC6608276

[B21] Connor C. E. Johnson K. O. (1992). Neural coding of tactile texture: comparison of spatial and temporal mechanisms for roughness perception. J. Neurosci. 12, 3414–3426. 10.1523/JNEUROSCI.12-09-03414.1992 1527586 PMC6575720

[B22] Costa L. D. F. Rodrigues F. A. Travieso G. Villas Boas P. R. (2007). Characterization of complex networks: a survey of measurements. Adv. Phys. 56, 167–242. 10.1080/00018730601170527

[B23] Crooks G. E. (1999). Entropy production fluctuation theorem and the nonequilibrium work relation for free energy differences. Phys. Rev. E 60, 2721–2726. 10.1103/PhysRevE.60.2721 11970075

[B24] Danos V. Oury N. (2013). Equilibrium and termination II: the case of petri nets. Math. Struct. Comput. Sci. 23, 290–307. 10.1017/S0960129512000126

[B25] Darwin C. (2003). On the Origin of Species by Means of Natural Selection. ed Carroll J. (Peterborough, ON: Broadview Press), 672.

[B26] De Vico Fallani F. Richiardi J. Chavez M. Achard S. (2014). Graph analysis of functional brain networks: practical issues in translational neuroscience. Philos. Trans. R. Soc. B Biol. Sci. 369:20130521. 10.1098/rstb.2013.0521 25180301 PMC4150298

[B27] Dehaene S. (2003). The neural basis of the Weber-Fechner law: a logarithmic mental number line. Trends Cogn. Sci. 7, 145–147. 10.1016/S1364-6613(03)00055-X 12691758

[B28] Demirtaş M. Deco G. (2018). Computational models of dysconnectivity in large-scale resting-state networks, in Computational Psychiatry: Mathematical Modeling of Mental Illness, eds Anticevic A. Murray J. D. (San Diego, CA: Elsevier Academic Press), 87–116. 10.1016/B978-0-12-809825-7.00004-3

[B29] Eguchi Y. Shimizu S. Tsujimoto Y. (1997). Intracellular ATP levels determine cell death fate by apoptosis or necrosis. Cancer Res. 57, 1835–1840. 9157970

[B30] Fairhall A. (2014). The receptive field is dead. Long live the receptive field? Curr. Opin. Neurobiol. 25, 9–12. 10.1016/j.conb.2014.02.001 24618227 PMC4043224

[B31] Feng J. Jost J. Qian M. (2007). Networks: From Biology to Theory, eds Feng J. Jost J. Qian M. (London: Springer). 10.1007/978-1-84628-780-0

[B32] Fodor J. A. (1983). The Modularity of Mind: An Essay on Faculty Psychology. Cambridge, MA: MIT Press, 145. 10.7551/mitpress/4737.001.0001

[B33] Friston K. (1997). Another neural code? Neuroimage 5, 213–220. 10.1006/nimg.1997.0260 9345550

[B34] Friston K. (2002). Beyond phrenology: what can neuroimaging tell us about distributed circuitry? Annu. Rev. Neurosci. 25, 221–250. 10.1146/annurev.neuro.25.112701.142846 12052909

[B35] Friston K. (2010). The free-energy principle: a unified brain theory? Nat. Rev. Neurosci. 11, 127–138. 10.1038/nrn2787 20068583

[B36] Gal A. Eytan D. Wallach A. Sandler M. Schiller J. Marom S. (2010). Dynamics of excitability over extended timescales in cultured cortical neurons. J. Neurosci. 30, 16332–16342. 10.1523/JNEUROSCI.4859-10.2010 21123579 PMC6634841

[B37] Gardner E. P. Johnson K. O. (2013). Sensory coding, in Principles of Neural Science, eds Kandel E. R. Schwartz J. H. Jessell T. (New York, NY: McGraw-Hill), 449–474. 10.1126/science.158.3799.399

[B38] Georgopoulos A. P. Schwartz A. B. Kettner R. E. (1986). Neuronal population coding of movement direction. Science 233, 1416–1419. 10.1126/science.3749885 3749885

[B39] Gilbert S. F. (2000a). Neural crest cells and axonal specificity, in Developmental Biology, ed Gilbert S. F. (Sunderland, MA: Sinauer Associates), 407–441.

[B40] Gilbert S. F. (2000b). The central nervous system and the epidermis, in Developmental Biology, ed Gilbert S. F. (Sunderland, MA: Sinauer Associates), 379–410.

[B41] Hardie D. G. (2011). Sensing of energy and nutrients by AMP-activated protein kinase. Am. J. Clin. Nutr. 93, 891S−896S. 10.3945/ajcn.110.001925 21325438

[B42] Hardie D. G. Ross F. A. Hawley S. A. (2012). AMPK: a nutrient and energy sensor that maintains energy homeostasis. Nat. Rev. Mol. Cell Biol. 13, 251–262. 10.1038/nrm3311 22436748 PMC5726489

[B43] Harris J. J. Jolivet R. Attwell D. (2012). Synaptic energy use and supply. Neuron 75, 762–777. 10.1016/j.neuron.2012.08.019 22958818

[B44] Herculano-Houzel S. (2011). Scaling of brain metabolism with a fixed energy budget per neuron: Implications for neuronal activity, plasticity and evolution. PLoS ONE. 6:e17514. 10.1371/journal.pone.0017514 21390261 PMC3046985

[B45] Hofmeyr J.-H. S. Cornish-Bowden A. (2000). Regulating the cellular economy of supply and demand. FEBS Lett. 476, 47–51. 10.1016/S0014-5793(00)01668-9 10878248

[B46] Horton A. C. Ehlers M. D. (2003). Neuronal polarity and trafficking. Neuron. 40, 277–295. 10.1016/S0896-6273(03)00629-9 14556709

[B47] Hovland C. I. (1960). Computer simulation of thinking. Am. Psychol. 15, 687–693. 10.1037/h0044165

[B48] Huang C.-W. Huang C.-C. Cheng J.-T. Tsai J.-J. Wu S.-N. (2007). Glucose and hippocampal neuronal excitability: Role of ATP-sensitive potassium channels. J. Neurosci. Res. 85, 1468–1477. 10.1002/jnr.21284 17410601

[B49] Huang Y. Rao R. P. N. (2011). Predictive coding. Wiley Interdiscip. Rev. Cogn. Sci. 2, 580–593. 10.1002/wcs.142 26302308

[B50] Hubel D. H. Wiesel T. N. (1962). Receptive fields, binocular interaction and functional architecture in the cat's visual cortex. J. Physiol. 160, 106–54. 10.1113/jphysiol.1962.sp006837 14449617 PMC1359523

[B51] Huttenlocher P. R. (1990). Morphometric study of human cerebral cortex development. Neuropsychologia 28, 517–527. 10.1016/0028-3932(90)90031-I 2203993

[B52] Hyder F. Patel A. B. Gjedde A. Rothman D. L. Behar K. L. Shulman R. G. (2006). Neuronal–glial glucose oxidation and glutamatergic–GABAergic function. J. Cereb. Blood Flow Metab. 26, 865–877. 10.1038/sj.jcbfm.9600263 16407855

[B53] Hyder F. Rothman D. L. Bennett M. R. (2013). Cortical energy demands of signaling and nonsignaling components in brain are conserved across mammalian species and activity levels. Proc. Natl. Acad. Sci. U.S.A. 110, 3549–3554. 10.1073/pnas.1214912110 23319606 PMC3587194

[B54] Jaynes E. T. (1965). Gibbs vs Boltzmann entropies. Am. J. Phys. 33, 391–398. 10.1119/1.1971557

[B55] Jekabsons M. B. Nicholls D. G. (2004). *In situ* respiration and bioenergetic status of mitochondria in primary cerebellar granule neuronal cultures exposed continuously to glutamate. J. Biol. Chem. 279, 32989–3000. 10.1074/jbc.M401540200 15166243

[B56] Jia Z. Collingridge G. L. (2017). Learning about synaptic GluA3. Neuron 93, 254–256. 10.1016/j.neuron.2017.01.004 28103474

[B57] Jimbo Y. Tateno T. Robinson H. P. C. (1999). Simultaneous induction of pathway-specific potentiation and depression in networks of cortical neurons. Biophys. J. 76, 670–678. 10.1016/S0006-3495(99)77234-6 9929472 PMC1300072

[B58] Kempter R. Gerstner W. Van Hemmen J. L. (1999). Hebbian learning and spiking neurons. Phys. Rev. E 59, 4498–4514. 10.1103/PhysRevE.59.4498

[B59] Klinger E. G. (2018). Approximate Bayesian Model Selection for Local Cortical Networks at Cellular Resolution. Available online at: https://mediatum.ub.tum.de/doc/1426522/file.pdf (accessed July 6, 2019).

[B60] Kostrzewa R. M. Segura-Aguilar J. (2003). Novel mechanisms and approaches in the study of neurodegeneration and neuroprotection. A review. Neurotox. Res. 5, 375–383. 10.1007/BF03033166 14715440

[B61] Lange S. C. Winkler U. Andresen L. Byhr,ø M. Waagepetersen H. S. Hirrlinger J.. (2015). Dynamic changes in cytosolic ATP levels in cultured glutamatergic neurons during NMDA-induced synaptic activity supported by glucose or lactate. Neurochem. Res. 40, 2517–2526. 10.1007/s11064-015-1651-9 26184116

[B62] Laughlin S. B. (2001). Energy as a constraint on the coding and processing of sensory information. Curr. Opin. Neurobiol. 11, 475–480. 10.1016/S0959-4388(00)00237-3 11502395

[B63] le Feber J. Stegenga J. Rutten W. L. C. (2010). The effect of slow electrical stimuli to achieve learning in cultured networks of rat cortical neurons. PLoS ONE 5:e8871. 10.1371/journal.pone.0008871 20111726 PMC2810341

[B122] Le Masson G. Przedborski P. Abbott L. F. (2014). A computational model of motor neuron degeneration. Neuron 83, 975–988. 10.1016/j.neuron.2014.07.001 25088365 PMC4167823

[B64] Lemak M. S. Voloshanenko O. Draguhn A. Egorov A. V. (2014). KATP channels modulate intrinsic firing activity of immature entorhinal cortex layer III neurons. Front. Cell. Neurosci. 8:255. 10.3389/fncel.2014.00255 25221474 PMC4145353

[B65] Lennie P. (2003). The cost of cortical computation. Curr. Biol. 13, 493–497. 10.1016/S0960-9822(03)00135-0 12646132

[B66] Lettvin J. Maturana H. McCulloch W. S. Pitts W. H. (1959). What the frog's eye tells the frog's brain, in Proceedings of the IRE. Available online at: http://ieeexplore.ieee.org/xpls/abs_all.jsp?arnumber=4065609 (accessed May 2, 2012).

[B67] Lorenz D. M. Jeng A. Deem M. W. (2011). The emergence of modularity in biological systems. Phys. Life Rev. 8, 129–160. 10.1016/j.plrev.2011.02.003 21353651 PMC4477837

[B68] Madhavan R. Chao Z. C. Potter S. M. (2007). Plasticity of recurring spatiotemporal activity patterns in cortical networks. Phys. Biol. 4, 181–193. 10.1088/1478-3975/4/3/005 17928657 PMC2577584

[B69] Magistretti P. J. (2006). Neuron – glia metabolic coupling and plasticity. J. Exp. Biol. 209, 2304–2311. 10.1242/jeb.02208 16731806

[B70] Magistretti P. J. Allaman I. (2015). A cellular perspective on brain energy metabolism and functional imaging. Neuron 86, 883–901. 10.1016/j.neuron.2015.03.035 25996133

[B71] Magistretti P. J. Allaman I. (2018). Lactate in the brain: from metabolic end-product to signalling molecule. Nat. Rev. Neurosci. 19, 235–249. 10.1038/nrn.2018.19 29515192

[B72] Marcaida G. Min ana M. D. Grisolía S. Felipo V. (1995). Lack of correlation between glutamate-induced depletion of ATP and neuronal death in primary cultures of cerebellum. Brain Res. 695, 146–150. 10.1016/0006-8993(95)00703-S 8556324

[B73] Marcaida G. Miñana M.-D. Grisolia S. Felipo V. (1997). Determination of intracellular ATP in primary cultures of neurons. Brain Res. Protoc. 1, 75–78. 10.1016/S1385-299X(96)00009-8 9385050

[B74] Marsland R. England J. (2018). Limits of predictions in thermodynamic systems: a review. Reports Prog. Phys. 81:016601. 10.1088/1361-6633/aa9101 28976362

[B75] Miller K. D. MacKay D. J. C. (1994). The role of constraints in hebbian learning. Neural Comput. 6, 100–126. 10.1162/neco.1994.6.1.100

[B76] Moser M.-B. Moser E. I. (2015). Where am I? Where am i going? Sci. Am. 314, 26–33. 10.1038/scientificamerican0116-26 26887193

[B77] Moxon K. A. Foffani G. (2015). Brain-machine interfaces beyond neuroprosthetics. Neuron 86, 55–67. 10.1016/j.neuron.2015.03.036 25856486

[B78] Mulas M. Massobrio P. (2010). A simulated neuro-robotic environment for bi-directional closed-loop experiments. Communication 1, 179–186. 10.2478/s13230-011-0004-x

[B79] Nabavi S. Fox R. Proulx C. D. Lin J. Y. Tsien R. Y. Malinow R. (2014). Engineering a memory with LTD and LTP. Nature 511, 348–352. 10.1038/nature13294 24896183 PMC4210354

[B80] Nicholls D. (2013). Bioenergetics, 4th Edn. London: Academic Press.

[B81] Nicolelis M. A. L. (2003). Brain-machine interfaces to restore motor function and probe neural circuits. Nat. Rev. Neurosci. 4, 417–422. 10.1038/nrn1105 12728268

[B82] Novellino A. D'Angelo P. Cozzi L. Chiappalone M. Sanguineti V. Martinoia S. (2007). Connecting Neurons to a mobile robot: an in vitro bidirectional neural interface. Comput. Intell. Neurosci. 2007, 1–13. 10.1155/2007/12725 PMC2266971 18350128

[B83] Olshausen B. A. Field D. J. (2005). How close are we to understanding v1? Neural Comput. 17, 1665–1699. 10.1162/0899766054026639 15969914

[B84] Otsu Y. Couchman K. Lyons D. G. Collot M. Agarwal A. Mallet J.-M.. (2015). Calcium dynamics in astrocyte processes during neurovascular coupling. Nat. Neurosci. 18, 210–218. 10.1038/nn.3906 25531572 PMC4651918

[B85] Ouldridge T. E. (2018). The importance of thermodynamics for molecular systems, and the importance of molecular systems for thermodynamics. Nat. Comput. 17, 3–29. 10.1007/s11047-017-9646-x 29576756 PMC5856891

[B86] Penfield W. (1965). Speech, perception and the uncommitted cortex, in Brain and Conscious Experience, ed Eccless J. C. (Berlin; Heidelberg: Springer), 217–237.

[B87] Penfield W. Boldrey E. (1937). Somatic motor and sensory representation in man. Brain, 389–443. 10.1093/brain/60.4.389

[B88] Perry E. K. McKeith I. Thompson P. Marshall E. Kerwin J. Jabeen S.. (1991). Topography, extent, and clinical relevance of neurochemical deficits in dementia of Lewy body type, Parkinson's disease, and Alzheimer's disease. Ann. N. Y. Acad. Sci. 640, 197–202. 10.1111/j.1749-6632.1991.tb00217.x 1723256

[B89] Pino O. Guílera G. Gómez-Benito J. Najas-García A. Rufián S. Rojo E. (2014). Neurodevelopment or neurodegeneration: Review of theories of schizophrenia. Actas Esp. Psiquiatr. 42, 185–195. Available online at: https://www.actaspsiquiatria.es/repositorio/16/90/ENG/16-90-ENG-185-195-865491.pdf (accessed July 6, 2019). 25017496

[B90] Pissadaki E. K. Bolam J. P. (2013). The energy cost of action potential propagation in dopamine neurons: clues to susceptibility in Parkinson's disease. Front. Comput. Neurosci. 7:13. 10.3389/fncom.2013.00013 23515615 PMC3600574

[B91] Potter W. B. O'Riordan K. J. Barnett D. Osting S. M. K. Wagoner M. Burger C.. (2010). Metabolic regulation of neuronal plasticity by the energy sensor AMPK. PLoS ONE. 5:e8996. 10.1371/journal.pone.0008996 20126541 PMC2813866

[B92] Ramamurthy S. Chang E. Cao Y. Zhu J. Ronnett G. V. (2014). AMPK activation regulates neuronal structure in developing hippocampal neurons. Neuroscience 259, 13–24. 10.1016/j.neuroscience.2013.11.048 24295634

[B93] Rangaraju V. Calloway N. Ryan T. A. (2014). Activity-driven local ATP synthesis is required for synaptic function. Cell 156, 825–835. 10.1016/j.cell.2013.12.042 24529383 PMC3955179

[B94] Reid R. C. Shapley R. M. (1992). Spatial structure of cone inputs to receptive fields in primate lateral geniculate nucleus. Nature 356, 716–718. 10.1038/356716a0 1570016

[B95] Riccomagno M. M. Kolodkin A. L. (2015). Sculpting neural circuits by axon and dendrite pruning. Annu. Rev. Cell Dev. Biol. 31, 779–805. 10.1146/annurev-cellbio-100913-013038 26436703 PMC4668927

[B96] Robbins P. (2010). Modularity of Mind. Standford Encycl. Phylosophy. Available online at: http://plato.stanford.edu/entries/modularity-mind/ (accessed October 19, 2011).

[B97] Robinson M. B. Jackson J. G. (2016). Astroglial glutamate transporters coordinate excitatory signaling and brain energetics. Neurochem. Int. 98, 56–71. 10.1016/j.neuint.2016.03.014 27013346 PMC4969184

[B98] Ruth Clemo H. Stein B. E. (1982). Somatosensory cortex: a new somatotopic representation. Brain Res. 235, 162–168. 10.1016/0006-8993(82)90207-4 7188320

[B99] Saez I. Duran J. Sinadinos C. Beltran A. Yanes O. Tevy M. F.. (2014). Neurons have an active glycogen metabolism that contributes to tolerance to hypoxia. J. Cereb. Blood Flow Metab. 34, 945–955. 10.1038/jcbfm.2014.33 24569689 PMC4050236

[B100] Schroeder C. E. Wilson D. A. Radman T. Scharfman H. Lakatos P. (2010). Dynamics of Active Sensing and perceptual selection. Curr. Opin. Neurobiol. 20, 172–176. 10.1016/j.conb.2010.02.010 20307966 PMC2963579

[B101] Searle J. R. (1980). Minds, brains, and programs. Behav. Brain Sci. 3, 417–424. 10.1017/S0140525X00005756

[B102] Sengupta B. Faisal A. A. Laughlin S. B. Niven J. E. (2013). The effect of cell size and channel density on neuronal information encoding and energy efficiency. J. Cereb. Blood Flow Metab. 33, 1465–1473. 10.1038/jcbfm.2013.103 23778164 PMC3764378

[B103] Sheppard G. M. (2016). Foundations of the Neuron Doctrine: 25th Anniversary Edition. Oxford: Oxford University Press. 10.1093/med/9780190259389.001.0001

[B104] Shulman R. G. Rothman D. L. Behar K. L. Hyder F. (2004). Energetic basis of brain activity: Implications for neuroimaging. Trends Neurosci. 27, 489–495. 10.1016/j.tins.2004.06.005 15271497

[B105] Silbey R. J. Alberty R. A. Moungi B. G. (2004). Physical Chemistry, 4th Edn. Hoboken, NJ: Wiley.

[B106] Sokoloff L. (2008). The physiological and biochemical bases of functional brain imaging. Cogn. Neurodyn. 2, 1–5. 10.1007/978-1-4020-8387-7_58 19003468 PMC2289249

[B107] Spratling M. W. (2008). Reconciling predictive coding and biased competition models of cortical function. Front. Comput. Neurosci. 2:4. 10.3389/neuro.10.004.2008 18978957 PMC2576514

[B108] Tarasov A. I. Griffiths E. J. Rutter G. A. (2012). Regulation of ATP production by mitochondrial Ca2+. Cell Calcium. 52, 28–35. 10.1016/j.ceca.2012.03.003 22502861 PMC3396849

[B109] Tateno T. Jimbo Y. (1999). Activity-dependent enhancement in the reliability of correlated spike timings in cultured cortical neurons. Biol. Cybern. 80, 45–55. 10.1007/s004220050503 9951397

[B110] Taylor C. (1978). Theory of edge detection. Proc. R. Soc. Lond. B Biol. Sci. 207, 187–217. 10.1098/rspb.1980.0020 6102765

[B111] Tessadori J. Bisio M. Martinoia S. Chiappalone M. (2012). Modular neuronal assemblies embodied in a closed-loop environment: toward future integration of brains and machines. Front. Neural Circuits 6:99. 10.3389/fncir.2012.00099 23248586 PMC3520178

[B112] Tessadori J. Venuta D. Kumar S. S. Bisio M. Pasquale V. Chiappalone M. (2013). Embodied neuronal assemblies: a closed-loop environment for coding and decoding studies, in 2013 6th International IEEE/EMBS Conference on Neural Engineering (NER) (San Diego, CA), 899–902. 10.1109/NER.2013.6696080

[B113] Toloe J. Mollajew R. Kügler S. Mironov S. L. (2014). Metabolic differences in hippocampal Rett neurons revealed by ATP imaging. Mol. Cell. Neurosci. 59, 47–56. 10.1016/j.mcn.2013.12.008 24394521

[B114] Trevisiol A. Saab A. S. Winkler U. Marx G. Imamura H. Möbius W.. (2017). Monitoring ATP dynamics in electrically active white matter tracts. Elife 6:e24241. 10.7554/eLife.24241 28414271 PMC5415357

[B115] Turrigiano G. (2012). Homeostatic synaptic plasticity: local and global mechanisms for stabilizing neuronal function. Cold Spring Harb. Perspect. Biol. 4:a005736–a005736. 10.1101/cshperspect.a005736 22086977 PMC3249629

[B116] Turrigiano G. Leslie K. R. Desai N. S. Rutherford L. C. Nelson S. B. (1998). Activity-dependent scaling of quantal amplitude in neocortical neurons. Nature. 391, 892–896. 10.1038/36103 9495341

[B117] Wang D. D. Bordey A. (2008). The astrocyte odyssey. Prog. Neurobiol. 86, 342–367. 10.1016/j.pneurobio.2008.09.015 18948166 PMC2613184

[B118] Wang S. Kobayashi K. Kogure Y. Yamanaka H. Yamamoto S. Yagi H.. (2018). Negative regulation of TRPA1 by AMPK in primary sensory neurons as a potential mechanism of painful diabetic neuropathy. Diabetes 67, 98–109. 10.2337/db17-0503 29025860

[B119] Weber B. Barros L. F. (2015). The astrocyte: powerhouse and recycling center. Cold Spring Harb. Perspect. Biol. 7:a020396. 10.1101/cshperspect.a020396 25680832 PMC4665076

[B120] Yuan Y. Huo H. Zhao P. Liu J. Liu J. Xing F.. (2018). Constraints of metabolic energy on the number of synaptic connections of neurons and the density of neuronal networks. Front. Comput. Neurosci. 12:91. 10.3389/fncom.2018.00091 30524259 PMC6256250

[B121] Yuste R. (2015). From the neuron doctrine to neural networks. Nat. Rev. Neurosci. 16, 487–497. 10.1038/nrn3962 26152865

