# Corrigendum: The Energy Homeostasis Principle: Neuronal Energy Regulation Drives Local Network Dynamics Generating Behavior

**DOI:** 10.3389/fncom.2020.599670

**Published:** 2020-10-29

**Authors:** Rodrigo C. Vergara, Sebastián Jaramillo-Riveri, Alejandro Luarte, Cristóbal Moënne-Loccoz, Rómulo Fuentes, Andrés Couve, Pedro E. Maldonado

**Affiliations:** ^1^Neurosystems Laboratory, Faculty of Medicine, Biomedical Neuroscience Institute, Universidad de Chile, Santiago, Chile; ^2^School of Biological Sciences, Institute of Cell Biology, University of Edinburgh, Edinburgh, United Kingdom; ^3^Cellular and Molecular Neurobiology Laboratory, Faculty of Medicine, Biomedical Neuroscience Institute, Universidad de Chile, Santiago, Chile; ^4^Motor Control Laboratory, Faculty of Medicine, Biomedical Neuroscience Institute, Universidad de Chile, Santiago, Chile; ^5^Department of Health Sciences, Faculty of Medicine, Pontificia Universidad Católica de Chile, Santiago, Chile

**Keywords:** homeostasis, energy, neuronal networks, behavior, emergent properties

Unfortunately, the first equation in our published article was missing the terms dividing the difference in Gibbs Free Energy (Equation 1). We deemed relevant to correct the equation to prevent any potential misunderstanding, and apologize for any inconvenience it may have caused.

In particular, the first equation should have been written as follow:

(1) $$\begin{array}{r} \frac{rate\left(X\to Y\right)}{rate\left(Y\to X\right)}=e^{-\frac{G\left(Y\right)-G\left(X\right)}{RT}} \end{array}$$

where R is Gas constant, and T the absolute temperature (Cannon and Baker, 2017). This equation describes the relation between the mean rates of any pair of reversible processes (from X to Y, and from Y to X) and the difference in Gibbs Free Energy between the states. Note that by definition the Gibbs Free Energy assumes Temperature to be constant.

The subsequent arguments presented in our article remain unaffected by this correction, as by talking about Gibbs Free Energy we were already assuming Temperature to be constant.

The authors apologize for this error and state that this does not change the analyzed variables or scientific conclusions of the article in any way.

## References

[B1] Cannon W. R. Baker S. E. (2017). Non-steady state mass action dynamics without rate constants: dynamics of coupled reactions using chemical potentials. Phys. Biol. 14:055003. 10.1088/1478-3975/aa7d80 28675379

